# Temporal allele frequency changes in large‐effect loci reveal potential fishing impacts on salmon life‐history diversity

**DOI:** 10.1111/eva.13690

**Published:** 2024-04-25

**Authors:** Antti Miettinen, Atso Romakkaniemi, Johan Dannewitz, Tapani Pakarinen, Stefan Palm, Lo Persson, Johan Östergren, Craig R. Primmer, Victoria L. Pritchard

**Affiliations:** ^1^ Organismal & Evolutionary Biology Research Programme, Faculty of Biological and Environmental Sciences University of Helsinki Helsinki Finland; ^2^ Institute of Biotechnology University of Helsinki Helsinki Finland; ^3^ Natural Resources Institute Finland (Luke) Oulu Finland; ^4^ Department of Aquatic Resources, Institute of Freshwater Research Swedish University of Agricultural Sciences Drottningholm Sweden; ^5^ Natural Resources Institute Finland (Luke) Helsinki Finland; ^6^ Department of Wildlife, Fish and Environmental Studies Swedish University of Agricultural Sciences Umeå Sweden; ^7^ Institute for Biodiversity & Freshwater Conservation University of the Highlands & Islands Inverness UK

**Keywords:** Baltic salmon, fisheries management, fisheries‐induced evolution, genetic stock identification, SNP, temporal genomics

## Abstract

Fishing has the potential to influence the life‐history traits of exploited populations. However, our understanding of how fisheries can induce evolutionary genetic changes remains incomplete. The discovery of large‐effect loci linked with ecologically important life‐history traits, such as age at maturity in Atlantic salmon (*Salmo salar*), provides an opportunity to study the impacts of temporally varying fishing pressures on these traits. A 93‐year archive of fish scales from wild Atlantic salmon catches from the northern Baltic Sea region allowed us to monitor variation in adaptive genetic diversity linked with age at maturity of wild Atlantic salmon populations. The dataset consisted of samples from both commercial and recreational fisheries that target salmon on their spawning migration. Using a genotyping‐by‐sequencing approach (GT‐seq), we discovered strong within‐season allele frequency changes at the *vgll3* locus linked with Atlantic salmon age at maturity: fishing in the early season preferentially targeted the *vgll3* variant linked with older maturation. We also found within‐season temporal variation in catch proportions of different wild Atlantic salmon subpopulations. Therefore, selective pressures of harvesting may vary depending on the seasonal timing of fishing, which has the potential to cause evolutionary changes in key life‐history traits and their diversity. This knowledge can be used to guide fisheries management to reduce the effects of fishing practices on salmon life‐history diversity. Thus, this study provides a tangible example of using genomic approaches to infer, monitor and help mitigate human impacts on adaptively important genetic variation in nature.

## 1 INTRODUCTION

Harvesting has the potential to influence life‐history diversity in exploited natural populations. Size‐selective fishing, for example, can induce changes in size and age at maturity of the target species (e.g. Allendorf et al., 2008; Czorlich et al., 2022; Heino et al., 2015). However, our understanding of when and how harvesting selection can induce evolutionary (i.e. genetic) changes in wild populations, as opposed to plastic (i.e. phenotypic) changes, remains incomplete (e.g. Heino et al., 2015).

Discoveries of large‐effect loci associated with ecologically important life‐history traits offer opportunities to study adaptive responses of populations to human‐induced selection, as these loci provide a direct link to monitor the genetic basis of functional traits. Temporal variation in allele frequencies at large‐effect loci can be monitored to infer potential evolutionary impacts of anthropogenic selective pressures on functional traits across decades or even centuries (e.g. Czorlich et al., 2018; Jensen et al., 2022; Thompson et al., 2019). Changes in allele frequencies of functionally important loci can substantially affect the adaptive potential of populations, and knowledge of such changes can provide important information to guide conservation and management actions for exploited populations (reviewed in Benham & Bowie, 2023; Waples et al., 2022).

Atlantic salmon (*Salmo salar*) is an anadromous fish targeted by both commercial and recreational fishing. It exhibits wide phenotypic diversity in several key life‐history traits, such as the number of years spent feeding at sea before returning to fresh water to spawn (“age at maturity” or “sea age”) and the timing of migrations between the marine and freshwater environments (e.g. Erkinaro et al., 2019). A major part of the variation in Atlantic salmon age at maturity is explained by a single locus spanning the *vgll3* (*vestigial‐like family member 3*) gene on chromosome 25 (Ayllon et al., 2015; Barson et al., 2015). Salmon carrying the *vgll3* allele associated with “later” or “older” age at maturity (“vgll3*L”) spend, on average, more years feeding at sea before returning to spawn than salmon with the “earlier” age at maturity allele (“vgll3*E”) (Barson et al., 2015). The gene exhibits sex‐dependent dominance, with male heterozygotes tending to mature younger while female heterozygotes tend to mature older. A second locus, associated with the *six6* gene on chromosome 9 (“six6*L”/“six6*E”), also contributes to the age at maturity phenotype in some salmon populations (e.g. Besnier et al., 2023; Sinclair‐Waters et al., 2022).

Salmon that mature at an older age are larger and have higher reproductive success than younger, and therefore smaller, maturing individuals, which makes them important for the overall abundance of salmon stocks (reviewed in Mobley et al., 2021). The proportion of old spawners can also positively correlate with the genetic diversity of salmon stocks (Vähä et al., 2007), underlining their importance for the overall reproduction and viability of populations (e.g. Birkeland & Dayton, 2005). Furthermore, large salmon are particularly sought‐after by commercial and recreational fishers and attract fishing tourism that can provide substantial economic benefits, especially to remote regions (e.g. Anderson & Lee, 2013; Myrvold et al., 2019; Pohja‐Mykrä et al., 2018; Pokki et al., 2018). Older‐maturing salmon have become rarer in many parts of the Atlantic salmon distribution (Chaput, 2012). Some rapid declines in mean age at maturity of salmon stocks have been accompanied by decreases in the frequency of the vgll3*L variant, demonstrating that the shift to younger average maturation age has, in these cases, been an evolutionary, rather than a plastic response (Czorlich et al., 2018, 2022; Jensen et al., 2022).

In general, older and larger wild Atlantic salmon return to freshwater to spawn earlier in the season than younger and smaller salmon, and female salmon return earlier in the season than males (e.g. Foldvik et al., 2024; Harvey et al., 2017; Jokikokko et al., 2004; Niemelä et al., 2006; Shearer, 1990). In addition, variation at the *six6* locus is known to influence return migration timing of Atlantic salmon independent of age at maturity phenotype, with the six6*L (associated with older age at maturity) allele being linked to later within‐season return timing in multiple populations (Cauwelier et al., 2018; Pritchard et al., 2018). Variation in the portfolio of age at maturity and timing of return migration occurs both across and within rivers: Atlantic salmon spawning in the upstream parts of river systems are often older and enter freshwater earlier during the spawning migration season than their downstream counterparts (e.g. Cauwelier et al., 2018; Miettinen et al., 2021; Stewart et al., 2002). The strong homing of Atlantic salmon back to their natal river areas means that this phenological variation may co‐occur with substantial population genetic substructuring. This implies that the timing of fishing during the spawning migration could have differential selective effects on different life‐history strategy components and genetic subpopulations of Atlantic salmon stocks (see Morita, 2019; Tillotson & Quinn, 2018).

Atlantic salmon in the Baltic Sea region of northern Europe are a mix of wild populations and hatchery‐produced stocks released as mitigation for the previous destruction of salmon rivers by hydropower development. There is strong phylogenetic differentiation among the salmon populations originating from the northernmost (Gulf of Bothnia), eastern and southern parts of the Baltic Sea basin (Säisä et al., 2005). The majority of Baltic salmon today are produced by wild rivers and hatcheries in the Gulf of Bothnia. After leaving fresh water, salmon from this region migrate to feeding grounds in the southern Baltic Sea, and when reaching age at maturity, they return during spring and summer along the coasts of the Gulf of Bothnia to their natal rivers for spawning.

Currently, the Gulf of Bothnia salmon stocks are targeted by commercial and recreational fisheries during their spawning migration, both along the coast and in the rivers (e.g. ICES, 2023; Jacobson et al., 2020; Whitlock et al., 2021). The Atlantic salmon sea fishery in the Baltic Sea is currently the largest in the world (ICES, 2023). The biggest contributor to this fisheries catch is the wild salmon stock that spawns in the interconnected Tornio (Torne in Swedish) and Kalix Rivers of Finland and Sweden (ICES, 2023). This stock experienced a strong bottleneck from the mid‐1970s to the mid‐1990s due to overfishing and other impacts, but has since rebounded (Romakkaniemi et al., 2003). Tornio‐Kalix salmon are genetically differentiated within, but not between, the two rivers, with upper river reaches forming one genetic cluster and lower regions of the rivers forming another (Miettinen et al., 2021). This population structure is associated with differences in the seasonal timing of both smolt emigration and adult return migration between the upstream and downstream parts of the system (Miettinen et al., 2021).

Salmon fishing regulations in the Baltic Sea are a controversial topic. Early‐season coastal fishing has been banned for several decades with the aim of assisting the recovery of wild salmon stocks. However, since 2017 Finland has advanced the start of the coastal fishing season to allow a limited amount of harvest in the early part of summer. As temporal selection resulting from seasonal fisheries openings and closures has potential to induce evolutionary changes in phenotypic traits of fish populations (Tillotson & Quinn, 2018), this legislative change has sparked discussions about whether it may allow excessive harvesting of large, older‐maturing salmon that return to the rivers earlier in the season, and thereby lead to a reduction in mean age at maturity of Baltic salmon stocks. This concern may be of particular relevance for the Tornio–Kalix salmon that are harvested primarily along the eastern (Finnish) side of the Gulf of Bothnia during their spawning migration (Whitlock et al., 2018).

Earlier studies of temporal and spatial variation in stock compositions of Baltic salmon exist (e.g. Koljonen, 2006; Koljonen & McKinnell, 1996), but to date, none have assessed the harvesting pressures faced by the Tornio–Kalix subpopulations as they migrate along the coasts of Gulf of Bothnia to spawn in the Tornio–Kalix River system. Furthermore, temporal or spatial variation in adaptively important loci, such as *vgll3 and six6*, has not been previously studied in Baltic salmon catches. Such genetic monitoring can provide crucially relevant information about stocks that are heavily affected by intensive fishing (e.g. Whitlock et al., 2018, 2021), especially if their different subcomponents face unequal selection pressures.

Here, we apply population genetic approaches to Baltic salmon scale samples collected over 93 years (1928–2020) from commercial and recreational fisheries to investigate whether coastal and river fishing of wild salmon target specific genetic subpopulations and life‐history components across the fishing season. This allowed us to address whether changes in the fishing season have the potential to alter the mean age at maturity of salmon stocks by the selective pressures they exert on large‐effect loci.

## 2 MATERIALS AND METHODS

### 2.1 Study area and sample collection

#### 2.1.1 Genetic baseline

**FIGURE 1:**
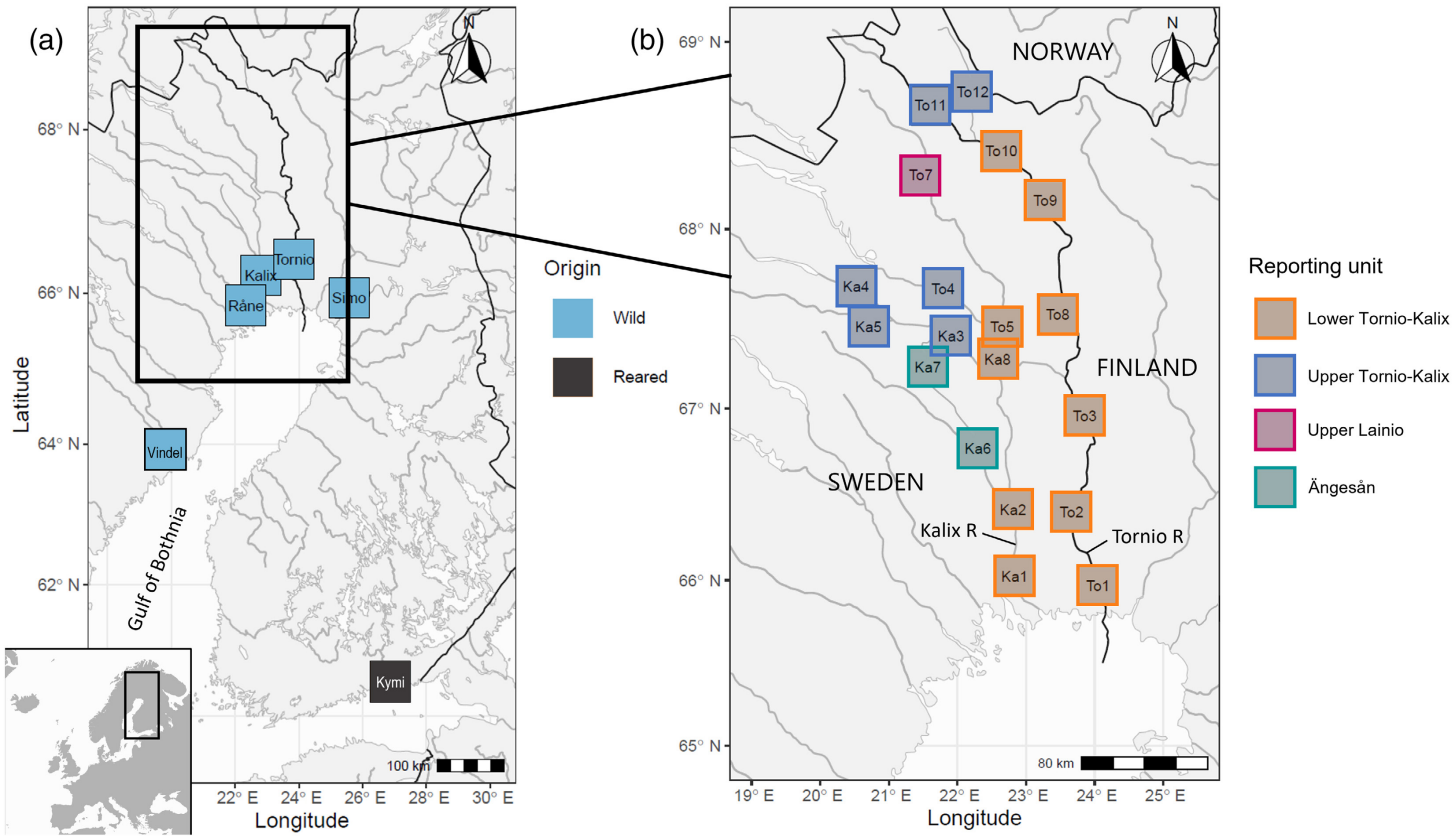
The geographic origin of (a) salmon stocks in the genetic baseline used for genetic stock identification in this study, and (b) sampling sites of wild baseline samples in the Tornio‐Kalix River system. The darker lines depict national borders, including the Tornio/Torne River that flows on the border of Finland and Sweden.

Full details of baseline sample collection, genotyping and analysis are provided in the Supplementary Methods and Results. The initial genetic baseline for this study comprised 496 wild salmon genotyped using a genome‐wide SNP array, divided into six reporting units based on analysis of population genetic structure: (1) lower Tornio–Kalix (*n* = 186; sample sites Ka1, Ka2, Ka8, To1, To2, To3, To5, To8, To9, To10), (2) upper Tornio–Kalix (*n* = 115; Ka3, Ka4, Ka5, To4, To11, To12), (3) upper Lainio (*n* = 20; To7), (4) Ängesån (*n* = 33; Ka6, Ka7), (5) Simo (*n* = 111) and (6) Råne (*n* = 31) Rivers (Figure 1).

We augmented this initial baseline with individuals from two additional reporting units, Vindel (*n* = 22) and Kymi (*n* = 19) Rivers (Figure 1), genotyped with a Genotyping‐in‐Thousands by sequencing (GT‐seq; Campbell et al., 2015) panel (see section 2.2 below). The Vindel River on the Swedish side of the Gulf of Bothnia supports a large wild salmon stock that along with neighboring, genetically similar rivers, may contribute a small number of individuals to the analyzed fisheries catch (Whitlock et al., 2018). The Kymi River hatchery stock originates from the Neva River and is, therefore, part of the eastern phylogenetic group of Baltic salmon from rivers in the Gulf of Finland and the south‐eastern Baltic Sea main basin (Säisä et al., 2005). Kymi was included in the baseline to “capture” any salmon belonging to that distinct lineage.

#### 2.1.2 Catch samples

We used tissue samples (dry scales) that had been collected from harvested Atlantic salmon as part of long‐term fisheries monitoring programs in the northern Baltic Sea region. These samples were from fish caught along the coast of the Gulf of Bothnia and within the Tornio and Kalix Rivers between 1928 and 2020. The sampling closely corresponded to the legal fishing seasons and within‐season variation in fishing efforts in each area. As our focus was on the impacts of fishing on wild populations, we discarded any hatchery‐origin fish (marked by adipose fin removal and/or identified via scale reading). We aimed to explore genetic and phenotypic variation in the wild salmon harvest across space and time within the fishing season, and long‐term changes across decades, rather than short‐term inter‐annual variation. Therefore, to maximize sample sizes, we combined samples from 2019 and 2020 to form a “contemporary” collection and combined samples collected in earlier years into nine “historical” collections, each representing a distinct period (years 1928–1930, 1937–1938, 1977–1981, 1989–1996, 2004–2006 and 2014–2016 for Tornio; and 1928–1931, 1937–1938 and 1962 for Kalix; Table 1; Table 2). We did not consider sex or age at maturity when selecting samples.

**TABLE 1:** Location and collection time of coastal wild salmon catch samples from 2019 to 2020 used in the study.

Area	Year	Sampling time span (dates)	*n* ^a^	Legal fishing season
Merikarvia (C1^b^)	2019	19 May – 17 July	93	1 May → 31 Dec.^c^
	2020	8 May – 15 July	92	
Luoto (C2^b^)	2019	28 May – 22 July	156	6 May → 31 Dec.^c^
	2020	29 May – 11 July	96	
Kemi River mouth (C3^b^)	2019	9 June – 3 July	96	16 May → 31 Dec.^c^
	2020	2 June – 2 July	82	
Tornio River mouth (C4^b^)	2019	14 June – 15 July	208	17 June → 31 Dec.^c^
	2020	11 June – 20 July	152	
Seskarö and Bergön, Sweden (C5^b^)	2020	18 June – 5 July	114	17 June – 5 July

*Note*: The legal fishing season refers to the time of the year when salmon fishing is allowed in each area.

^a^ Samples in the filtered dataset.

^b^ Location on Figure 2.

^c^ Spawning migration and consequently the salmon fishing season at the Gulf of Bothnian coast practically ends by August.

**TABLE 2:** Tornio/Torne and Kalix River samples from 1928 to 2020 used in the study.

Area	Time span (years)	Time span (dates)	*n* ^a^	Mean sea age	Note
Kalix River	1928–1931	12 June – 22 Aug.	91	2.38	Fishing method unknown. Catch date and specific location for some samples unknown
	1937–1938	27 June – 5 Aug.	70	2.40	
	1962	3 June – 26 July	83	2.32	
Torne River, within Sweden	1928–1930	17 June – 14 Aug.	87	2.54	
	1937–1938	2 June – 23 Aug.	99	2.28	
Tornio River, within Finland	1977–1981	10 June – 31 Aug.	81	1.68	Mostly other than rod angling catches
	1989–1996	12 June – 2 Sept.	105	2.14	Mostly rod angling catches
	2004–2006	26 May – 15 Aug.	137	2.23	Only rod angling catches
	2014–2016	1 June – 31 Aug.	208	2.08	
	2019–2020		695	1.95	

^a^ Samples in the filtered dataset.

##### Coastal catch samples 2019–2020

**FIGURE 2:**
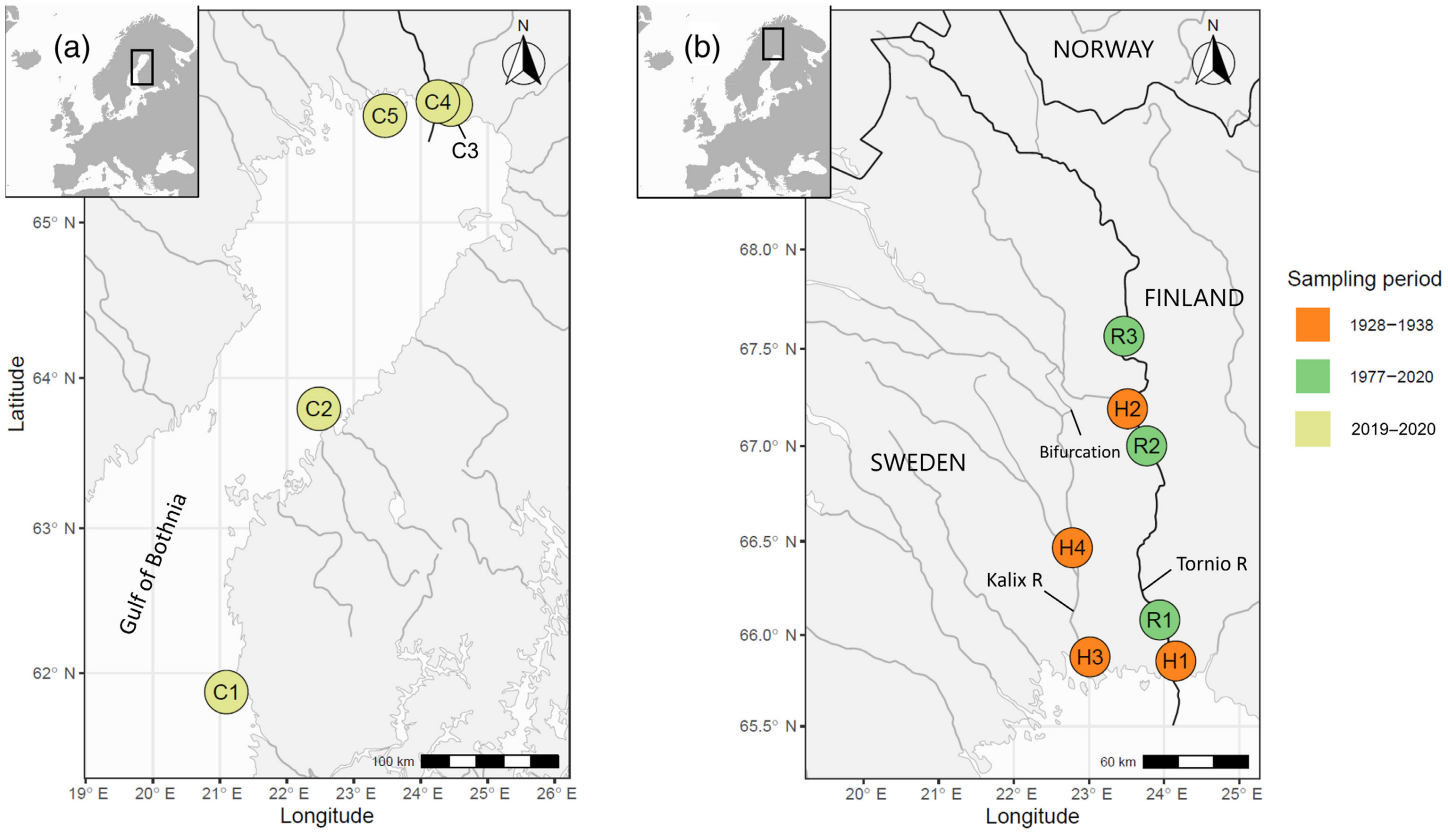
Collection of catch samples from (a) the coast of Gulf of Bothnia (years 2019–2020), and (b) from the Tornio and Kalix Rivers (years 1928–2020). Fishing areas on the maps: (a) see Table 1 for names of coastal fishing areas; (b) R1 = Tornio River Downstream area, R2 = Pello‐Lappea, R3 = Kihlanki, H1 = Kiviranta, H2 = Kengisfors, H3 = Kalix River Downstream area, H4 = Rödupp. The darker lines depict national borders, including the Tornio/Torne River that flows on the border of Finland and Sweden. Note that the bifurcation linking Tornio and Kalix (marked with a line) allows fish to access Tornio headwaters via the lower Kalix mainstem and vice versa.

Samples from contemporary coastal mixed‐stock salmon fisheries were collected in five areas of the Gulf of Bothnia during the fishing seasons of 2019 and 2020 (Figure 2; Table 1). The sampling locations represented the main salmon fishing areas in the Gulf of Bothnia, where wild‐spawned fish make up 70%–75% of the annual harvest (Pakarinen et al., 2022). Samples were obtained using salmon trap nets by Finnish and Swedish commercial fishers, who collected the samples across the entire season that they were fishing. All samples were taken within the legal national fishing seasons, apart from 25 samples from site C4 that were taken for research purposes by a permitted fisher before the season opening. We analyzed all sampled wild individuals except from site C1, from where we used a subset of individuals selected to represent the entire fishing season. In total, we genotyped 1098 coastal catch samples.

##### Tornio River catch samples 2019–2020

We also examined the genetic composition of the contemporary recreational fishing harvest in the Tornio River. Samples were collected in 2019–2020 from the three main fishing areas on the Finnish side of the Tornio River (R1/Downstream: 0–40 km upstream from the river mouth; R2/Pello‐Lappea: 120–180 km from the river mouth; R3/Kihlanki: 240–280 km from the river mouth; Figure 2). All samples were collected by anglers using rod and reel, which has been the most common fishing method within the river since the 1970s. Fishing in the Downstream area (R1) mostly takes place in the early season and the fish caught in the area are primarily migrating to spawning grounds further upstream in the Tornio–Kalix system (Miettinen et al., 2021). In the Pello‐Lappea (R2) and Kihlanki areas (R3), fishing occurs throughout the season and targets both local spawners and fish migrating to spawning grounds further upstream. We selected samples (*n* = 698) to be representative of the total wild catch throughout the seasons. Samples from these three river stretches can be assumed to represent the majority of the Tornio River salmon river catches.

##### Tornio and Kalix River catch samples 1928–2016

To examine temporal changes in the genetic composition of the Tornio‐Kalix River catches over time, we took advantage of archived tissue samples held in Finland and Sweden. We selected a representative subset (*n* = 1012) of all available samples (*n* > 10,000), and limited our analysis to samples collected between 26 May and 2 September to avoid temporal biases among years (Table 2).

*Tornio River, Finland (1977–2016)*: Samples from the Finnish side of the Tornio River were only available from the 1970s onward. We selected a subset of samples distributed across sites R1, R2 and R3, collected as described above (Table 2, total samples successfully genotyped from 1977 to 2016 = 531). Between the late 1970s and the early 2000s, the Finnish Tornio River was stocked with hatchery‐produced juvenile salmon of Tornio River origin (Romakkaniemi et al., 2003). To limit our analysis to wild‐spawned fish, we excluded samples from any fish that had been marked by adipose fin removal and/or exhibited scale characteristics typical of stocked fish (Hiilivirta et al., 1998). The adipose fin was not removed during the first years of the stocking practice, and we therefore excluded all samples that could originate from those cohorts.

*Torne and Kalix Rivers, Sweden (1928–1962)*: Samples from the Swedish side of the Tornio/Torne River were collected from two named areas (Kengisfors and Kiviranta) during the years 1930 (*n* = 50) and 1937–1938 (*n* = 101), respectively. In addition, 59 samples were collected from unknown Tornio/Torne locations during 1928–1929. Samples from the Kalix River during the years 1928–1938 were collected from four named sites (Kamlunge, Månsbyn, Näsbyn, Rödupp) that were collapsed into two areas based on geographic proximity. In addition, 86 samples were collected from unknown Kalix River locations in 1962 (Figure 2; Table 2). No information was available on the catch method for these samples. A part of these samples was used in the temporal genetic study of Östergren et al. (2021).

##### Phenotypic data

Catch location, date, phenotypic sex and (since the 1970s) the presence/absence of adipose fin had been recorded for adult samples at the time of capture. The method of phenotypic sexing was generally not stated. We also had data for all adults on age at maturity, defined as the number of winters at sea before first spawning (“sea age”, number of “sea‐winters”), from scale readings performed following international guidelines specific for Atlantic salmon (ICES, 2011). If the recorded sex and genetic sex (see below) were discordant, we used genetic sex in our analysis.

### 2.2 DNA extractions and genotyping‐by‐sequencing

We used QuickExtract™ Solution (Lucigen) to extract DNA from the 1977–2020 Tornio River catch samples and the 2019–2020 coastal catch samples from Sweden. The rest of the coastal samples were extracted by the Natural Resources Institute Finland (Luke, Jokioinen), using the QIAGEN DNeasy Blood & Tissue Kit. DNA was extracted from the 1928–1962 Tornio and Kalix River samples either using the Chemagic™ 360 instrument (PerkinElmer, at University of Helsinki) or the QIAGEN QIAsymphony robot and QIAsymphony DNA extraction kit (at the Swedish University of Agricultural Sciences; SLU), following the manufacturer's instructions. We tested DNA concentrations and integrity with NanoDrop (Thermo Fisher Scientific), Qubit (Thermo Fisher Scientific) and/or agarose gel electrophoresis.

We genotyped and sexed 2803 coastal and river catch samples and the 41 additional baseline samples (Vindel/Kymi) using a 229 SNP genotyping‐by‐sequencing panel (GT‐seq) developed and optimized for this study. This panel includes markers linked to the adaptively important large‐effect loci *vgll3* (*vgll3*_*top*_ marker; vgll3*L = C/G; vgll3*E = A/T) and *six6* (*six6*_*top*_ marker; six6*L = A/T; six6*E = C/G) (Barson et al., 2015) plus a marker on the male‐specific *sdY* locus (Yano et al., 2013). Full details on the SNP panel development and genotyping are provided in the Supplementary Methods & Results.

Using *vcftools* 0.1.17 (Danecek et al., 2011), we removed genotypes with a genotype quality of <15 (*‐‐minGQ 15*) and a read depth of <8 (*‐‐minDP 8*). Using PLINK v1.90 (Chang et al., 2015), we then filtered out SNPs and individuals with >30% missing data (*‐‐geno 0.3 ‐‐mind 0.3*) from this genotyping‐by‐sequencing dataset.

### 2.3 Genetic stock identification

#### 2.3.1 Units for mixed stock analysis

We first estimated the overall stock composition of the total contemporary (2019–2020) coastal and river catch collections and the nine historical river catch collections. For the three most recent sampling periods (2019–2020, 2014–2016 and 2004–2006), for which we had larger numbers of samples, we also qualitatively explored trends in the stock composition of catches across the fishing season. To do this, we divided the total samples from each fishing location into four quartiles, which represented the early, early‐mid, late‐mid and late parts of the fishing season at that location (Table S1). Quartiles were defined by ordering the samples from each location by catch day, splitting them into four consecutive equally sized groups, and then adjusting the quartile boundaries so that fish caught on the same day were not split between quartiles. Note that, using this approach, each location‐specific quartile contains an approximately equal number of fish but the date boundaries for the quartiles are different at each fishing location. We took this approach rather than splitting samples by predefined temporal boundaries (e.g. catch month) to avoid estimating mixture proportions from very different sample sizes. We did not investigate within‐season trends in stock composition for site C5, from which the sample size was small and the fishing season short.

For 2019–2020, we also estimated how many individual salmon from each reporting group were caught across the season at each location. We obtained cumulative total catch numbers of wild salmon (available for 1‐day intervals in the coastal areas managed by Sweden, 5‐day intervals in the coastal areas managed by Finland, and 15–16‐day intervals in the Tornio River) and used these to infer daily catch numbers. We then combined these with our stock composition estimates (see below) to estimate the total number of fish from each genetic reporting unit caught in the early, early‐mid, late‐mid and late seasonal quartiles at each location.

#### 2.3.2 Performance of SNP panel

We tested the performance of the SNP panel with 166 SNPs for GSI by using a “leave‐one‐out” population re‐assignment procedure implemented in R package *rubias* (Moran & Anderson, 2019; R Core Team, 2021). We used the proportion of baseline individuals re‐assigned to their correct reporting unit as a measure of SNP panel performance.

#### 2.3.3 Genetic stock analysis

We performed mixed stock analysis separately for all catch subsamples (fishing areas, quartiles and time points) using a maximum likelihood approach implemented in the R package *rubias*. We used the genetic baseline described above and default options of a uniform prior and 200 burn‐in, followed by 2000 MCMC replicates.

#### 2.3.4 Population of origin

We used information from the mixed stock analyses to assign individuals to their most likely population of origin. Individuals with a probability (PofZ) < 0.7 of originating from any baseline population were considered unassigned (following Aykanat et al., 2020).

#### 2.3.5 Phenotypic variation across Tornio–Kalix subpopulations

To examine life‐history variation across Tornio–Kalix subpopulations, we calculated mean age at maturity and capture date across salmon assigned by *rubias* to each of the subpopulations in the entire catch dataset (1928–2020).

### 2.4 Patterns of phenotypic variation associated with maturation‐linked loci

#### 2.4.1 Association of *vgll3* and *six6* genotypes with age at maturity

We investigated the relationship between sex, *vgll3 and six6* genotypes and age at maturity across the entire catch dataset using multinomial logistic regression implemented in *nnet 7.3.19* in R (Venables & Ripley, 2002). As only 39 fish had a sea age of four sea‐winters or older, we used three age at maturity categories: 1, 2 and 3+. The initial model included additive effects of *vgll3* and *six6* (coded as count of *L* alleles) plus a dominance effect for each locus (coded as 1 for heterozygotes, and 0 otherwise). To account for the known sex‐specific dominance at *vgll3*, we modelled males and females separately. We assessed the significance of each effect term by comparing the likelihoods of a full model and a reduced model lacking that term, using likelihood ratio tests (*lmtest 0.9.40* in R, Zeileis & Hothorn, 2002). We removed all non‐significant terms from the full model and repeated this process with the subsequent reduced models to arrive at the final model.

#### 2.4.2 Patterns of phenotypic and genotypic variation across the fishing season

##### Variation in sex and age at maturity across the fishing season

We investigated the relationship of sex and age at maturity with salmon catch date, coded as days since 1 May, within the total 2019–2020 fisheries catch. We implemented a general mixed model using *lme4 1.1.34* (Bates et al., 2015) in R, with sex, age at maturity and their interaction as fixed factors and year and fishing location as random factors. We selected the model terms based on their ecological relevance and statistical significance. We visually assessed the normality of residuals for the models with the *qqnorm* function in *lme4*, and homoscedasticity by using the *plot* function in R.

##### Variation in *vgll3* and *six6* allele frequencies across the fishing season

We investigated the relationship between vgll3*L allele count, six6*L allele count and salmon catch date. We initially examined these across the entire catch dataset. To investigate in detail the potential impact of the current fishing regime, we also performed separate analyses for the coastal and river catches from 2019–2020. Using *lme4* in R, we first applied a general linear mixed model with catch date as the dependent variable, vgll3*L and six6*L allele count as fixed factors, and fishing area and year as random factors. To ask whether *vgll3* and/or *six6* effects on catch date were entirely mediated through their effects on the age at maturity phenotype, we repeated this analysis with the addition of the fixed factors sex, age at maturity and their interaction. We selected the model terms based on their ecological relevance and statistical significance. We visually assessed the normality of residuals for the models with the *qqnorm* function in *lme4*, and homoscedasticity by using the *plot* function in R. To account for the possibility that our results could be biased by including different stocks with different allele frequencies and migration timing, we repeated all analyses including only individuals assigned by *rubias* to the large lower Tornio–Kalix population.

To illustrate and visually examine the change in vgll3*L and six6*L allele frequencies across the seasons in the 2019–2020 fisheries catches, we used *ggplot2* to plot them graphically and used *geom_smooth()* to fit a generalised additive model (GAM) across the data separately for the river and coastal catches, and also for each fishing area. We similarly illustrated and visually examined these within‐season changes in the nine historical collections (Table 2).

#### 2.4.3 Changes in *vgll3* and *six6* allele frequencies across years

To examine whether any SNPs exhibited unusually high variation in allele frequency across the 93‐year sampling period, which could suggest temporally varying selection, we used OutFLANK_0.2 (Whitlock & Lotterhos, 2015) to identify SNPs with unusually high *F*_ST_ among the seven contemporary or historical collections from the Tornio River (Table 2). We applied default parameters and considered SNPs with *q* < 0.05 to be significant *F*_ST_ outliers. To illustrate and visually examine the changes in vgll3*L and six6*L allele frequencies in the Tornio River catches over time, we fit a GAM across the data (using *ggplot2* and *geom_smooth()* as above).

## 3 RESULTS

### 3.1 Genotyping quality control

Out of the 2803 catch samples and 229 SNPs genotyped with the GT‐seq panel, 56 individuals and 59 SNPs were removed due to exceeding the missing data threshold, and one individual due to unusually high heterozygosity suggesting sample contamination. One SNP was removed due to mismatches between genotypes inferred by array genotyping and genotypes inferred by genotyping‐by‐sequencing. The final dataset comprised 2745 catch samples and 41 additional baseline samples (Vindel and Kymi Rivers) genotyped at 166 baseline SNPs, *vgll3*_*top*_, *six6*_*top*_ and the *sdY* marker. Mean genotyping success across the 168 autosomal SNPs was 95.3%. Phenotypic sex was discordant with genotypic sex based on the *sdY* marker in 261 (9.5%) catch samples.

To generate the genetic baseline for the genetic stock identification (GSI) of the catch samples, we extracted the genotypes for the 166 SNPs from the SNP array dataset, and added the genotyping‐by‐sequencing genotypes for two additional baseline populations (Vindel and Kymi). We also removed seven baseline individuals from the Tornio–Kalix system that, on the basis of population structure analysis (details in Supplementary Methods and Results), appeared to originate from locations further upstream in the system than where they were collected.

### 3.2 Genetic stock identification of Baltic salmon catches

The estimated self‐assignment accuracy of samples from the baseline populations using the 166 retained GSI SNPs ranged from 87.9% (Ängesån tributary in the Kalix River) to 100% (Kymi, Råne and Vindel Rivers) (Table S2).

Mixture analysis inferred that the majority of wild salmon caught in the contemporary (2019–2020) coastal and Tornio River fishery originated from the lower Tornio–Kalix genetic cluster (coastal fishery: 75.9%; river fishery: 80.5%). An additional 2.8% and 17.5% of the coastal catches, and 8.5% and 10.5% of the river catches originated from the upper Lainio and other upper reaches of the Tornio–Kalix system, respectively (Table S3; Figure S1). Stock proportions in the historical (1928–2016) collections were relatively constant, but in the Tornio River there was an overall decrease in the proportion of upper Tornio–Kalix salmon over time (Figure S1).

**FIGURE 3:**
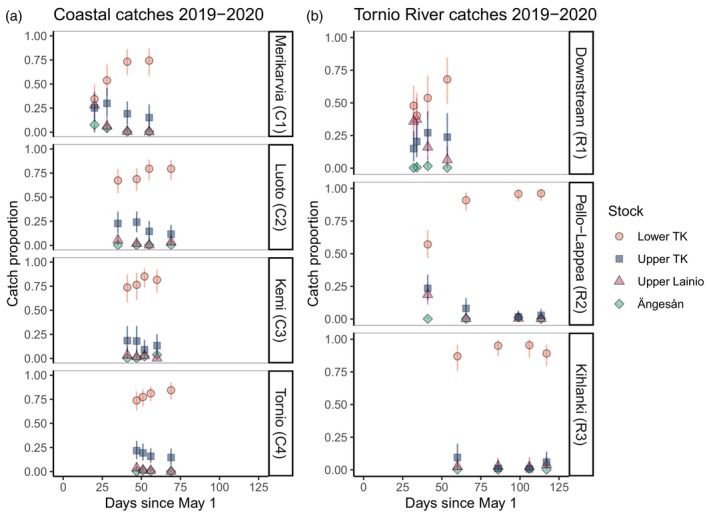
Estimated stock proportions of wild salmon catches from (a) the coastal fishing areas and (b) the Tornio River during fishing seasons 2019–2020. The catches were split temporally into quartiles (the four points per stock and area) based on the number of genetic samples. The points represent median quartile days (i.e. median catch date of individuals in each quartile), counted as days since May 1 (see Table S1). The error bars denote 95% credible intervals. For illustrative purposes, estimated proportions of Kymi, Råne, Simo and Vindel River stocks are not shown. TK refers to Tornio‐Kalix.

Visual inspection indicated that for 2019–2020, the proportion of catch originating from the upper Tornio–Kalix and upper Lainio decreased across the fishing season in the coastal fishing areas (Merikarvia, Luoto, Kemi River mouth, Tornio River mouth), and in the Downstream (R1) and Pello‐Lappea (R2) fishing areas in the Tornio River (Figure 3a). Overall, salmon originating from upper Tornio–Kalix and upper Lainio were particularly well‐represented (c. 45%) in the Downstream catches from the Tornio River (mostly taken early in the fishing season), whereas by far most of the catches from the more upstream Pello‐Lappea and Kihlanki (R3) areas (mainly taken later in the season) originated from lower Tornio–Kalix (Figure 3b). The pattern of increasing proportions of salmon originating from the lower Tornio–Kalix over the season was evident also in the Tornio River catches from 2004–2006 and 2014–2016 (Figure S2).

#### 3.2.1 Phenotypic variation across Tornio–Kalix subpopulations

Visual inspection of the entire catch dataset indicated that salmon assigned to the upper parts of the Tornio–Kalix tended to be caught earlier in the season than salmon assigned to the lower Tornio–Kalix cluster. Salmon originating from the upper Lainio were caught particularly early and had an old age at maturity (Table 3).

**TABLE 3:** Phenotypic and life‐history loci variation across individuals assigned to Tornio‐Kalix subpopulations in the dataset of catches from 1928 to 2020.

Subpopulation	Sex	*n*	Sea age (mean + SD)	Catch date (since may 1; mean + SD)	vgll3*L frequency (mean + SD)	six6*L frequency (mean + SD)
Lower Tornio‐Kalix	Female	1150	2.28 + 0.58	64.23 + 23.59	0.55 + 0.36	0.80 + 0.29
	Male	889	1.82 + 0.82	72.57 + 25.17	0.55 + 0.36	0.80 + 0.29
	Total	2068	2.08 + 0.73	67.72 + 24.58	0.55 + 0.36	0.80 + 0.29
Upper Tornio‐Kalix	Female	237	2.08 + 0.43	52.07 + 17.68	0.56 + 0.36	0.47 + 0.37
	Male	163	1.84 + 0.74	57.92 + 19.93	0.63 + 0.36	0.46 + 0.36
	Total	405	1.99 + 0.59	54.53 + 18.81	0.58 + 0.36	0.47 + 0.36
Upper Lainio	Female	57	2.28 + 0.53	40.46 + 18.99	0.82 + 0.28	0.86 + 0.25
	Male	43	2.38 + 0.70	44.77 + 23.51	0.83 + 0.29	0.83 + 0.26
	Total	102	2.33 + 0.60	41.79 + 21.18	0.82 + 0.28	0.85 + 0.25
Ängesån	Female	10	1.90 + 0.32	52.90 + 22.88	0.20 + 0.35	0.75 + 0.26
	Male	5	1.40 + 0.89	48.80 + 12.62	0.40 + 0.22	0.80 + 0.27
	Total	15	1.73 + 0.59	51.53 + 19.65	0.27 + 0.32	0.77 + 0.26

### 3.3 Patterns of phenotypic variation associated with maturation‐linked loci

#### 3.3.1 Association of *vgll3* and *six6* genotypes with age at maturity

**FIGURE 4:**
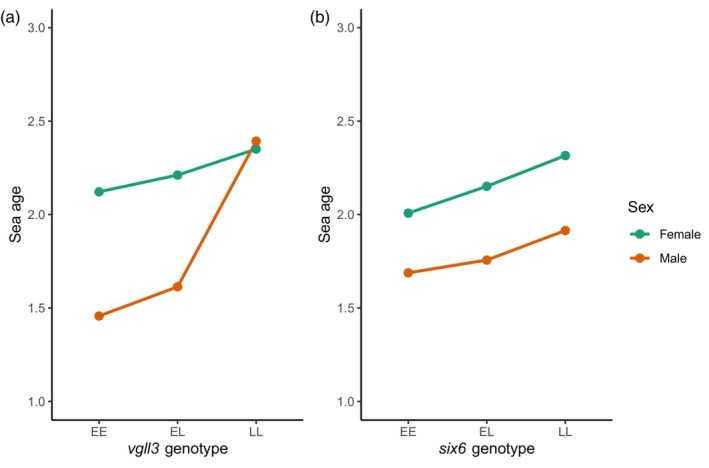
Relationship between mean age at maturity of salmon (i.e. sea age, as number of winters spent at sea before first spawning) and genotypes of the (a) *vgll3* and (b) *six6* genes, analysed across the entire Baltic salmon catch dataset from 1928 to 2020. E and L represent the alleles associated with early and late maturation, respectively.

When applying a multinomial logistic regression model with the entire catch dataset, we observed significant additive effects of *vgll3* and *six6* on age at maturity in both males and females (Figure 4; Table S4). We additionally observed a significant *vgll3* dominance effect in males, but not in females. No *six6* dominance effect was seen for either sex (Table S4a). Information on the final fitted models is provided in Table S4b.

#### 3.3.2 Patterns of phenotypic and genotypic variation across the fishing season

Females were caught earlier in the season than males in both coastal (*t* = 4.62, *p* < 0.001) and river (*t* = 6.70, *p* < 0.001) fisheries, as well as across the entire catch dataset in 2019–2020 (*t* = 8.65, *p* < 0.001; Table S4c). We found a significant relationship between *vgll3* genotype and catch date when examining the entire catch dataset (from 1928–2020; *t* = −11.64, *p* < 0.001), and when examining the 2019–2020 coastal (*t* = −5.59, *p* < 0.001) and river (*t* = −6.59, *p* < 0.001) fisheries separately. We also observed a significant association between *six6* genotype and catch date in the entire catch dataset (from 1928–2020; *t* = 4.98, *p* < 0.001) and the 2019–2020 coastal dataset (*t* = 2.09, *p* = 0.037), with six6*L being associated with a later catch date (Table S4d). When accounting for the mixed stock structure of our dataset by applying the same models as above only to individuals assigned to the lower Tornio–Kalix, the significant effect of *vgll3* on catch date persisted in the entire catch dataset (*t* = −9.25, *p* < 0.001) and the 2019–2020 coastal (*t* = −5.33, *p* < 0.001) and river (*t* = −4.48, *p* < 0.001) datasets, but the *six6* effect was no longer significant (Table S4d).

**FIGURE 5:**
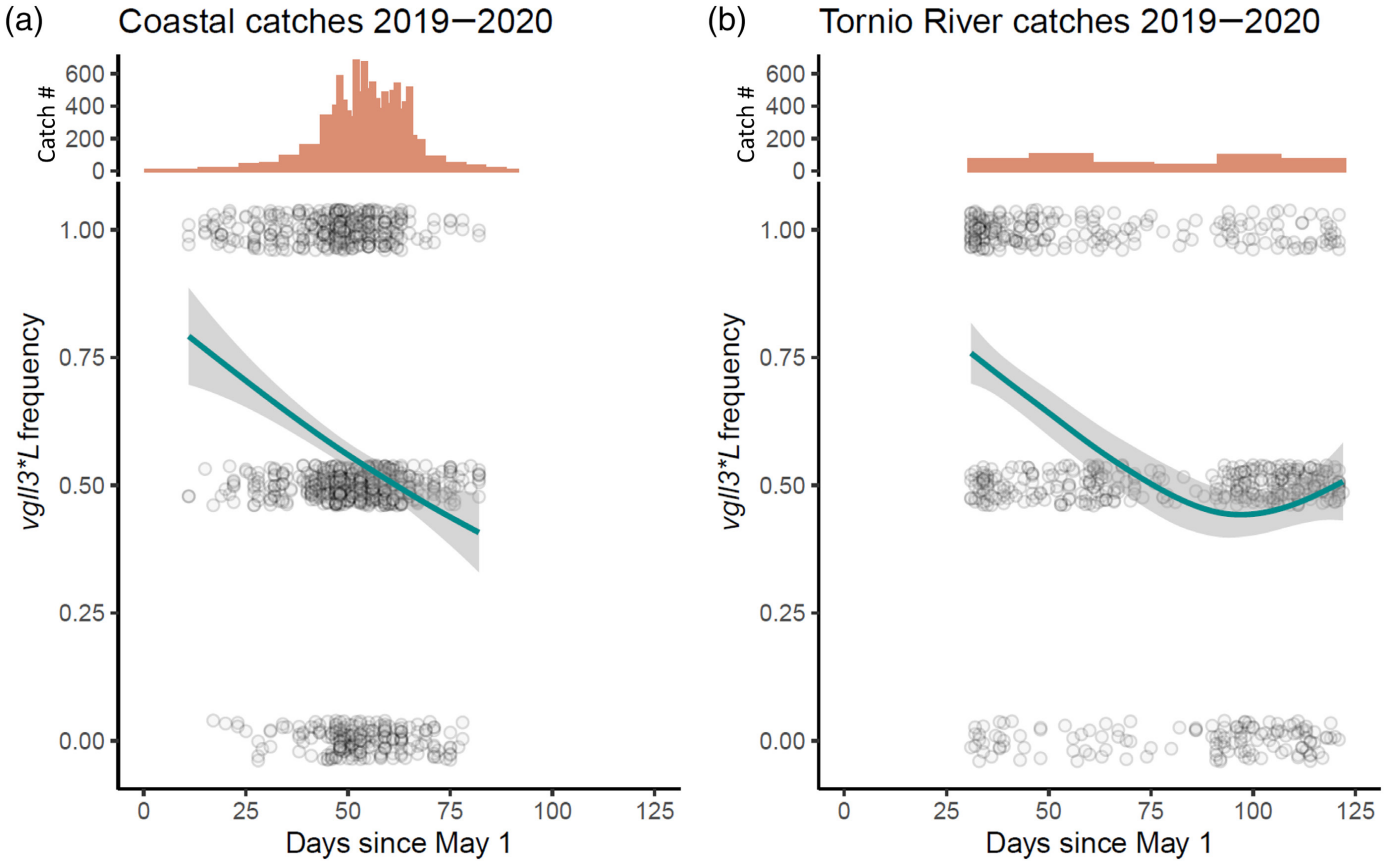
vgll3*L (associated with older age at maturity) allele frequency in (a) coastal wild salmon catches, and in (b) Tornio River catches during 2019–2020. The lines depict a relationship between vgll3*L and catch date, fitted with a GAM (smoother used: y ~ s(x, bs = “cs”)), whereas the grey area around the lines illustrates the uncertainty of the fitted relationship (95% confidence intervals). The histograms above show the estimated daily catch sizes (number of salmon caught per day) in these areas for the duration of the fishing season. The data points represent the *vgll3* genotype of individual samples. The points are jittered on the y‐axis to aid figure interpretation. TK refers to Tornio‐Kalix.

Visual exploration of the data confirmed that the frequency of vgll3*L strongly decreased over the main fishing season (of 13 weeks) in catches from each coastal fishing area in 2019–2020 (Figure 5a; Figure S3), apart from the area managed by Sweden (C5), for which the fishing season was extremely short (2½ weeks; not shown). When the frequency of vgll3*L was examined in combination with estimates of daily total catches from the different coastal fishing areas managed by Finland (C1 to C4), we found that relatively few salmon (in terms of absolute numbers of salmon caught, compared to later in the season) were caught early in the season when the variant was most frequent in the catches (Figure 5a; Figure S3).

The frequency of vgll3*L in the 2019–2020 Tornio River catches also strongly decreased during the fishing season in each fishing area (Figure 5b; Figure S4). In the Downstream area (R1), most salmon were caught early in the season when fish with the vgll3*L allele were most abundant in the catches. In the areas further upstream (Pello‐Lappea, R1; Kihlanki, R2), more salmon were caught later in the season, when the vgll3*L allele was less abundant in the catches. In contrast to *vgll3*, no consistent pattern was observed for *six6* across the different fishing areas (Figure S5, different areas not shown).

A similar decline in vgll3*L allele frequency across the fishing season was observed for nearly all historical time points (Figures S6 and S7), whereas six6*L frequency either increased across the season or showed no trend (Figures S6 and S7).

We found that *vgll3* and *six6* genotypes had a significant effect on catch date also when sex and sea age were controlled for. For all datasets examined, except for the Tornio River catches 2019–2020 with only fish assigned to the lower Tornio–Kalix, vgll3*L was associated with an advance in catch date and six6*L with a delay in catch date (Table S4e). This effect was seen most clearly when plotting the change in *vgll3* and *six6* allele frequencies over time within two‐sea‐winter fish (the most frequent age category in the catches): vgll3*L allele frequency generally declined over the season, while six6*L frequency generally increased (Figure S8).

#### 3.3.3 Long‐term temporal genetic variation in maturation‐linked loci

**FIGURE 6:**
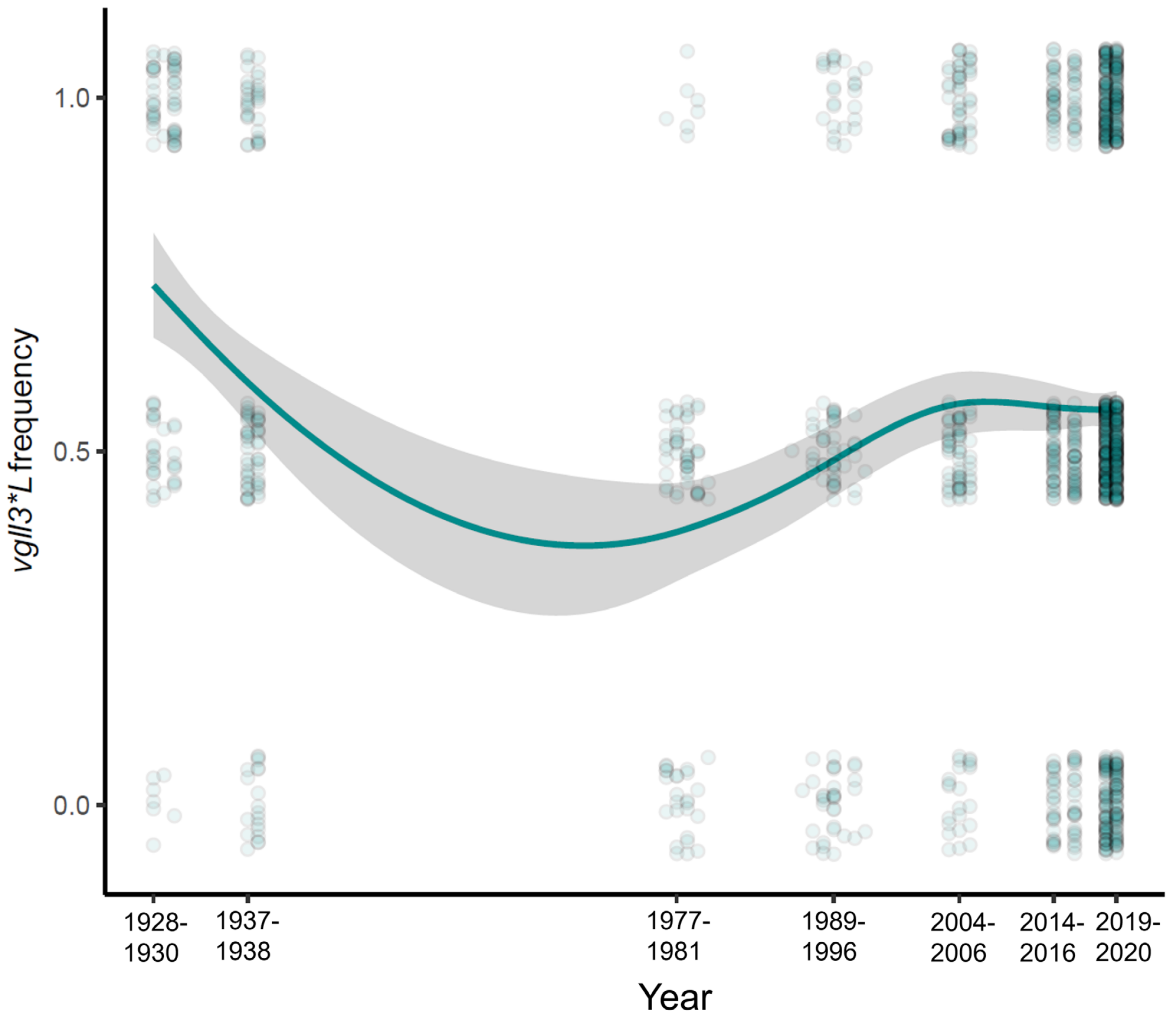
Long‐term changes in vgll3*L allele frequency in the Tornio/Torne River salmon catches from 1928 to 2020. The line depicts a relationship between vgll3*L and sampling year, fitted with a GAM (smoother used: y ~ s(x, bs = “cs”)), whereas the grey area around the lines illustrates the uncertainty of the fitted relationship (95% confidence intervals). The data points represent the *vgll3* genotype of individual samples. The points are jittered on the *y*‐axis to aid figure interpretation.

The vgll3*L frequency in the Tornio River salmon catches appeared to fluctuate considerably over the 93‐year time span of the available data (Figure 6; see results from OutFLANK analysis below). The variant was particularly frequent in the samples from 1928 to 1930, and rarest during the late 1970s and early 1980s. After that, the variant became more frequent until the early 2000s (2004–2006), after which changes in its frequency were relatively small. In contrast, the frequency of the six6*L allele appeared to increase over time (Figure S9).

One SNP was removed from the OutFLANK analysis due to failing the heterozygosity threshold (*H*_e_ < 0.1). Analysis with OutFLANK across time points identified two significant *F*_ST_ outliers among the 167 remaining SNPs: *vgll3*, with the highest *F*_ST_ across time points (*q* < 0.0025), and one baseline SNP (*q* < 0.028; on chromosome 9, c. 11.3 Mb apart from six6_top_) (Figure S10). *Six6* exhibited the fourth‐highest *F*_ST_ across time points but was not a significant outlier (*q* = 0.236).

## 4 DISCUSSION

Using genotyping‐by‐sequencing on 2745 wild Atlantic salmon caught by commercial and recreational fisheries in the northern Baltic Sea, we discovered that fishing in the early season targeted a genetic variant strongly linked with older age of maturation (vgll3*L on chromosome 25; Ayllon et al., 2015; Barson et al., 2015). This suggests that temporally varying fishing mortality within the season, due to for example earlier openings to the season, has the potential to cause evolutionary changes in a key life‐history trait and its diversity in the salmon populations targeted by these fisheries. We could also confirm that subpopulations spawning upstream in the largest Baltic salmon river complex are more frequent in early than late‐season catches (Miettinen et al., 2021), further highlighting the potential impact of seasonal fishing timing on these populations. This knowledge, and the novel genetic tools that we present in this article, can be used to guide management to reduce the potential deleterious effects of fishing practices on salmon populations and their life‐history diversity. Overall, this study provides a tangible example of using genomic approaches to infer, monitor and help mitigate human impacts on adaptively important genetic variation in nature.

Furthermore, this study improved our ability to identify Baltic salmon subpopulations from mixed stock fishery catches, and therefore to quantify selective pressures extorted on them by both coastal and river fisheries. This improves the selection of tools available to effectively manage and conserve different components of these wild salmon stocks.

### 4.1 Temporally varying fishing mortality can induce life‐history changes

We found that salmon caught in the early season possessed higher frequencies of vgll3*L than late‐season catches, and so also exhibited greater sea age, in both coastal and river fisheries. This indicates that heavy fishing mortality on salmon migrating early in the summer has the potential to cause evolutionary changes in the age at maturity of Baltic salmon stocks: mean reproductive age and size of fish may decrease if fishing strongly and continuously targets larger individuals (e.g. Conover & Munch, 2002; Jørgensen et al., 2007; Uusi‐Heikkilä et al., 2015). Using specific genetic markers to monitor or manage potential harvest‐induced evolution is still possible in only few species. In Atlantic salmon, the observed relationship between catch date and vgll3*L can be used to improve predictions of evolutionary responses of salmon to temporally varying harvesting pressures.

We also confirmed an additional effect of the genetic variant six6*L on Atlantic salmon age at maturity, as well as an association of six6*L with catch date that was in the opposite direction to vgll3*L. Corresponding with the observation that six6*L is strongly associated with later river mouth catch date within an Atlantic salmon age class in Scotland (Cauwelier et al., 2018), and also in the Teno River of northern Finland/Norway (Pritchard et al., 2018), we found that six6*L frequency was positively associated with catch date also when the effect of vgll3*L was accounted for. However, the realised change in six6*L frequency across the fishing season was weak, making any effect of changing fisheries regimes on *six6* difficult to predict. The observed small increase in the frequency of the six6*L allele between 1928 and 2020 may reflect the decreasing proportions of upper Tornio‐Kalix fish in the catches, rather than directional selection at this locus.

Interestingly, both *vgll*3 and *six*6 were significantly associated with catch date when sex and age at maturity were accounted for, but these associations were in different directions. In the case of *vgll3*, this suggests that the association of vgll3*L with earlier catch date is not solely mediated through its effect on age at maturity; fish of the same age class tend to be caught earlier if they carry more vgll3*L alleles. This indicates that strong early season fishing has the potential to cause evolutionary changes towards younger maturation age by selecting against vgll3*L even if older salmon would be left unharvested. The opposing effects of *vgll3* and *six6* on catch date, a good proxy for the timing of the return spawning migration, suggest that there could be selection on the joint *vgll3*‐*six6* allele spectrum to optimise return timing in wild Atlantic salmon populations, in addition to the selection that is acting to optimise age at maturity. This could explain observations that Atlantic salmon populations differing in their age at maturity portfolio may also exhibit large differences in six6*L allele frequency (e.g. Barson et al., 2015).

The vgll3*L allele has become rarer over recent decades in Atlantic salmon catches of the Teno and Eira Rivers of Finland and Norway, respectively (Czorlich et al., 2018; Jensen et al., 2022). For both rivers, this genetic change was accompanied with a reduction in mean age at maturity. Czorlich et al. (2022) inferred direct and indirect effects of fishing to underlie this rapid evolutionary change in the Teno, while habitat modifications were suggested to be behind the change in the Eira (Jensen et al., 2022). Here, we found that allele frequencies of *vgll3* in the Tornio River catches over 93 years fluctuated more than any of the 167 other SNPs genotyped. The frequency of vgll3*L was highest in the oldest samples (1928–1930) and lowest in the late 1970s‐early 1980s. However, we caution that although our catch samples from different years were similarly distributed across the season and had a relatively similar mixed stock composition, we cannot completely rule out an influence of different catch dates, locations or stocks on this observation. Some uncertainty about fishing methods for older samples, meaning that fish could have been harvested in a size‐selective manner, is another possible source of bias. Nevertheless, the observed long‐term fluctuations are reasonably concordant with historical age structure changes of the Tornio‐Kalix River salmon catches, that have likely been affected by changes in fishing (e.g. Kallio‐Nyberg et al., 2014; Karlsson & Karlström, 1994; Romakkaniemi et al., 2003), among other factors (e.g. climatic variation; Huusko & Hyvärinen, 2012). Salmon caught in the Tornio–Kalix in the early 1900s were, on average, notably larger and older than today (e.g. Järvi, 1938; Kallio‐Nyberg et al., 2014). Offshore fishing intensified especially between the 1960s and 1980s (Karlsson & Karlström, 1994) and consequently increased the mortality of salmon spending multiple years at sea (Romakkaniemi et al., 2003). The average size and sea age of Tornio River catches strongly increased in the 1990s (e.g. Romakkaniemi et al., 2003), likely due to a combination of reduced offshore fishing, strong restrictions on coastal fishing including the enforcement of an early summer ban, and high survival of salmon at sea owing to abundant prey stocks and warm winters (between 1982 and 1992) (Karlsson & Karlström, 1994; Romakkaniemi et al., 2003). We observe similar changes in our multi‐year dataset (Table 2).

The vgll3*L frequency has remained relatively stable in the 2000s, and our results do not suggest that it would have become rarer in the catches since 2017, when coastal salmon fishing managed by Finland in the Gulf of Bothnia has been, to a limited extent, allowed to start as soon as environmental conditions permit. Due to restrictions on both fishing effort and catch quotas, the intensity of fishing during the “advanced” part of the season (i.e. allowed since 2017) in 2019–2020 may not have been strong enough (Pakarinen et al., 2022) to have caused a directly detectable vgll3*L reduction in the Tornio River catches in these years. However, due to the long generation time of late‐maturing salmon in this region, evaluating potential evolutionary changes due to a regulation change in 2017 requires assessment over a longer time period than is possible with this dataset. Thus, we recommend continued genetic monitoring of subsequent generations of salmon to evaluate the potential evolutionary impact of the “advanced” early season fishing.

We also found that early season fishing, both on the coast of Gulf of Bothnia and in the Tornio River, caught relatively more salmon originating from upstream sites of the Tornio–Kalix system than late season fishing. This is in line with a previous microsatellite study that analysed stock composition of 2009–2010 adult salmon catches in the Tornio River (Miettinen et al., 2021), and concordant with findings of seasonal migration timing differences within rivers in other salmon lineages (e.g. Niemelä et al., 2006; Stewart et al., 2002; Vähä et al., 2011). Notably, salmon assigned to the upper Lainio genetic cluster were frequent only in the earliest catches. Salmon originating from this subpopulation and other upstream parts of the Tornio–Kalix were also, on average, older than catches originating from the lower reaches. These results support earlier views on within‐river differences in migration behaviour and age structure in the Tornio–Kalix system, and suggest that subpopulations spawning in the upper river reaches may be particularly vulnerable to strong early season fishing (Miettinen et al., 2021). Particular concern for these populations is justified because their longer migrations and longer life cycle (due to an older smolt age; Miettinen et al., 2021) compared to downstream populations predispose them to higher fishing and natural mortality.

### 4.2 Significance of results to salmon management

Diversity in life‐history traits, such as age at maturity or migration timing, may buffer populations against environmental changes, and can reduce variability among years in the numbers of fish that return to spawn and can be sustainably harvested from salmonid populations (e.g. Carvalho et al., 2023; Connors et al., 2022; Cordoleani et al., 2021; Gharrett et al., 2013; Hoelzel et al., 2019; Schindler et al., 2010). Fishing has been shown to cause evolutionary changes in the mean age and size at maturity of salmon populations (Czorlich et al., 2022). As the restoration of genetic traits altered by fishing is a slow natural process, proactive preservation of this kind of ecologically important diversity is recommended (e.g. Enberg et al., 2009). Our results suggest that to safeguard variation in age at maturity and migration timing in salmon stocks, the potential evolutionary impacts of temporally varying fishing pressures need to be carefully considered in regulating fisheries (Mobley et al., 2021). Preserving this kind of phenological variation within and among salmon populations by avoiding harvest‐induced selection can promote the populations' resilience to climate change (Kovach et al., 2015), help them endure stochastic environments and minimise the need for fisheries closures (Schindler et al., 2010).

Our genetic analysis of coastal catches from 2019–2020 provides evidence that restrictions on early summer coastal salmon fishing are important to prevent the excessive targeting of the earliest migrating salmon, that mainly originate from populations in upstream river areas. The results also demonstrated that the vgll3*L variant was always more frequent in catches from each coastal area during the “advanced” fishing season, compared to any later time point. In addition, compared to later in the summer, catches from the “advanced” season were more often females, which are particularly important for the reproduction and recruitment of salmon stocks (e.g. Jonsson & Jonsson 2011). Thus, we argue that the current temporal restrictions of the coastal fisheries are important and should not be relaxed, as they help to conserve the viability of the upstream populations in particular, as well as life‐history variation and subsequently the adaptive potential and long‐term resiliency of the Tornio–Kalix stock. This is, in turn, expected to maintain the genetic diversity of Baltic salmon as a whole (Kurland et al., 2023).

Salmon fishing in the Downstream area of the Tornio River appeared to target the upstream subpopulations and vgll3*L variant particularly strongly compared to other studied fishing areas. Angling along the Tornio River has increased markedly following a relatively recent recovery of the river's salmon stock (Palm et al., 2023), and fishing in the Downstream area is more concentrated in the early summer than in other areas. Due to the differences in regulation and conditions of coastal and river fishing, the river fishing can harvest salmon that have reached the river before the initiation of full‐scale coastal fishing. According to sonar monitoring at a site c. 100 km upstream from the Tornio River mouth (Kattilakoski), only very few salmon migrate through the lowest river reach before the start of river fishing on June 1st (Palm et al., 2023). This and our results suggest that Downstream fishing in the Tornio River currently mostly capitalizes on the stock component that the coastal fishing restrictions protect. Thus, regulating the Downstream fishing could be a powerful additional measure in conserving the old and upstream‐spawning salmon (see Harvey et al., 2017; Quinn et al., 2006), and should be considered to protect these subpopulations and the genetic variants underlying old age at maturity and/or early migration timing. Reducing fishing mortality on the upstream subpopulations could also help to enhance the overall production and population size of the entire river system. To further assess the area‐specific impact of harvesting on the Tornio–Kalix subpopulations, future studies could compare the genetic composition and vgll3*L frequencies of smolts emigrating from the river system to the proportional genetic composition of adult catches.

### 4.3 Conclusions

Knowledge of the genetic basis of age at maturity in Atlantic salmon (Ayllon et al., 2015; Barson et al., 2015) combined with genotyping an extensive time series of catch samples provided us with a rare opportunity to infer possible evolutionary impacts of fishing on wild salmon populations. Our results suggest that variation in seasonal timing of fishing has the potential to cause evolutionary changes in key life‐history traits of Atlantic salmon. Most notably, we found the frequency of the *vgll3* variant linked with older maturation to decrease in fishing catches over the season, which suggests that strong early season fishing could lead to a reduction in mean reproductive age and size of salmon.

## CONFLICT OF INTEREST STATEMENT

The authors declare no conflict of interest.

## Supporting information


Data S1.

## Data Availability

Data and codes used in the analyses of this study are available in the Zenodo repository: https://doi.org/10.5281/zenodo.10782524.

## References

[eva13690-bib-0003] Allendorf, F. W. , England, P. R. , Luikart, G. , Ritchie, P. A. , & Ryman, N. (2008). Genetic effects of harvest on wild animal populations. Trends in Ecology & Evolution, 23(6), 327–337. 10.1016/j.tree.2008.02.008 18439706

[eva13690-bib-0004] Anderson, L. E. , & Lee, S. T. (2013). Untangling the recreational value of wild and hatchery Salmon. Marine Resource Economics, 28(2), 175–197. 10.5950/0738-1360-28.2.175

[eva13690-bib-0005] Aykanat, T. , Rasmussen, M. , Ozerov, M. , Niemelä, E. , Paulin, L. , Vähä, J.‐P. , Hindar, K. , Wennevik, V. , Pedersen, T. , Svenning, M.‐A. , & Primmer, C. R. (2020). Life‐history genomic regions explain differences in Atlantic salmon marine diet specialization. The Journal of Animal Ecology, 89, 2677–2691. 33460064 10.1111/1365-2656.13324

[eva13690-bib-0006] Ayllon, F. , Kjærner‐Semb, E. , Furmanek, T. , Wennevik, V. , Solberg, M. F. , Dahle, G. , Taranger, G. L. , Glover, K. A. , Sällman Almén, M. , Rubin, C. J. , Edvardsen, R. B. , & Wargelius, A. (2015). The vgll3 locus controls age at maturity in wild and domesticated Atlantic Salmon (*Salmo salar* L.) males. PLoS Genetics, 11(11), 1–15. 10.1371/journal.pgen.1005628 PMC4638356 26551894

[eva13690-bib-0007] Barson, N. J. , Aykanat, T. , Hindar, K. , Baranski, M. , Bolstad, G. H. , Fiske, P. , Jacq, C. , Jensen, A. J. , Johnston, S. E. , Karlsson, S. , Kent, M. , Moen, T. , Niemelä, E. , Nome, T. , Næsje, T. F. , Orell, P. , Romakkaniemi, A. , Sægrov, H. , Urdal, K. , … Primmer, C. R. (2015). Sex‐dependent dominance at a single locus maintains variation in age at maturity in salmon. Nature, 528(7582), 405–408. 10.1038/nature16062 26536110

[eva13690-bib-0008] Bates, D. , Mächler, M. , Bolker, B. M. , & Walker, S. C. (2015). Fitting linear mixed‐effects models using lme4. Journal of Statistical Software, 67(1), 1–48. 10.18637/jss.v067.i01

[eva13690-bib-0009] Benham, P. M. , & Bowie, R. C. K. (2023). Natural history collections as a resource for conservation genomics: Understanding the past to preserve the future. The Journal of Heredity, 114(4), 367–384. 36512345 10.1093/jhered/esac066

[eva13690-bib-0010] Besnier, F. , Skaala, Ø. , Wennevik, V. , Ayllon, F. , Utne, K. R. , Fjeldheim, P. T. , Andersen‐Fjeldheim, K. , Knutar, S. , & Glover, K. A. (2023). Overruled by nature: A plastic response to environmental change disconnects a gene and its trait. Molecular Ecology, 33, e16933. 10.1111/mec.16933 36942798

[eva13690-bib-0011] Birkeland, C. , & Dayton, P. K. (2005). The importance in fishery management of leaving the big ones. Trends in Ecology & Evolution, 20(7), 283–289. 10.1016/j.tree.2005.03.015 16701393

[eva13690-bib-0012] Campbell, N. R. , Harmon, S. A. , & Narum, S. R. (2015). Genotyping‐in‐thousands by sequencing (GT‐seq): A cost effective SNP genotyping method based on custom amplicon sequencing. Molecular Ecology Resources, 15(4), 855–867. 10.1111/1755-0998.12357 25476721

[eva13690-bib-0013] Carvalho, P. G. , Satterthwaite, W. H. , O'Farrell, M. R. , Speir, C. , & Palkovacs, E. P. (2023). Role of maturation and mortality in portfolio effects and climate resilience. Canadian Journal of Fisheries and Aquatic Sciences, 80(6), 924–941. 10.1139/cjfas-2022-0171

[eva13690-bib-0014] Cauwelier, E. , Gilbey, J. , Sampayo, J. , Stradmeyer, L. , & Middlemas, S. J. (2018). Identification of a single genomic region associated with seasonal river return timing in adult Scottish Atlantic salmon (*Salmo salar*), using a genome‐wide association study. Canadian Journal of Fisheries and Aquatic Sciences, 75(9), 1427–1435. 10.1139/cjfas-2017-0293

[eva13690-bib-0015] Chang, C. C. , Chow, C. C. , Tellier, L. C. A. M. , Vattikuti, S. , Purcell, S. M. , & Lee, J. J. (2015). Second‐generation PLINK: Rising to the challenge of larger and richer datasets. GigaScience, 4(1), 1–16. 10.1186/s13742-015-0047-8 25722852 PMC4342193

[eva13690-bib-0016] Chaput, G. (2012). Overview of the status of Atlantic salmon (*Salmo salar*) in the North Atlantic and trends in marine mortality. ICES Journal of Marine Science, 69(9), 1538–1548. 10.1093/icesjms/fst048

[eva13690-bib-0017] Connors, B. M. , Siegle, M. R. , Harding, J. , Rossi, S. , Staton, B. A. , Jones, M. L. , Bradford, M. J. , Brown, R. , Bechtol, B. , Doherty, B. , Cox, S. , & Sutherland, B. J. G. (2022). Chinook salmon diversity contributes to fishery stability and trade‐offs with mixed‐stock harvest. Ecological Applications, 32(8), 1–17. 10.1002/eap.2709 36131546

[eva13690-bib-0018] Conover, D. O. , & Munch, S. B. (2002). Sustaining fisheries yields over evolutionary time scales. Science, 297(5578), 94–96. 10.1126/science.1074085 12098697

[eva13690-bib-0019] Cordoleani, F. , Phillis, C. C. , Sturrock, A. M. , Fitzgerald, A. M. , Malkassian, A. , Whitman, G. E. , Weber, P. K. , & Johnson, R. C. (2021). Threatened salmon rely on a rare life history strategy in a warming landscape. Nature Climate Change, 11, 982–988. 10.1038/s41558-021-01186-4

[eva13690-bib-0020] Czorlich, Y. , Aykanat, T. , Erkinaro, J. , Orell, P. , & Primmer, C. R. (2018). Rapid sex‐specific evolution of age at maturity is shaped by genetic architecture in Atlantic salmon. Nature Ecology & Evolution, 2(11), 1800–1807. 10.1038/s41559-018-0681-5 30275465 PMC6322654

[eva13690-bib-0021] Czorlich, Y. , Aykanat, T. , Erkinaro, J. , Orell, P. , & Primmer, C. R. (2022). Rapid evolution in salmon life history induced by direct and indirect effects of fishing. Science, 376(6591), 420–423. 10.1126/science.abg5980 35201899

[eva13690-bib-0022] Danecek, P. , Auton, A. , Abecasis, G. , Albers, C. A. , Banks, E. , DePristo, M. A. , Handsaker, R. E. , Lunter, G. , Marth, G. T. , Sherry, S. T. , McVean, G. , Durbin, R. , & 1000 Genomes Project Analysis Group . (2011). The variant call format and VCFtools. Bioinformatics, 27(15), 2156–2158. 10.1093/bioinformatics/btr330 21653522 PMC3137218

[eva13690-bib-0023] Enberg, K. , Jørgensen, C. , Dunlop, E. S. , Heino, M. , & Dieckmann, U. (2009). Implications of fisheries‐induced evolution for stock rebuilding and recovery. Evolutionary Applications, 2(3), 394–414. 10.1111/j.1752-4571.2009.00077.x 25567888 PMC3352485

[eva13690-bib-0024] Erkinaro, J. , Czorlich, Y. , Orell, P. , Kuusela, J. , Falkegård, M. , Länsman, M. , Pulkkinen, H. , Primmer, C. R. , & Niemelä, E. (2019). Life history variation across four decades in a diverse population complex of Atlantic salmon in a large subarctic river. Canadian Journal of Fisheries and Aquatic Sciences, 76(1), 42–55. 10.1139/cjfas-2017-0343

[eva13690-bib-0025] Foldvik, A. , Ulvan, E. M. , & Næsje, T. (2024). Optimal timing of return migration in Atlantic salmon. Fish and Fisheries, 1–12. 10.1111/faf.12816

[eva13690-bib-0026] Gharrett, A. J. , Joyce, J. , & Smoker, W. W. (2013). Fine‐scale temporal adaptation within a salmonid population: Mechanism and consequences. Molecular Ecology, 22(17), 4457–4469. 10.1111/mec.12400 23980763

[eva13690-bib-0027] Harvey, A. C. , Tang, Y. , Wennevik, V. , Skaala, Ø. , & Glover, K. A. (2017). Timing is everything: Fishing‐season placement may represent the most important angling‐induced evolutionary pressure on Atlantic salmon populations. Ecology and Evolution, 7(18), 7490–7502. 10.1002/ece3.3304 28944033 PMC5606871

[eva13690-bib-0028] Heino, M. , Díaz Pauli, B. , & Dieckmann, U. (2015). Fisheries‐induced evolution. Annual Review of Ecology, Evolution, and Systematics, 46(1), 461–480. 10.1146/annurev-ecolsys-112414-054339

[eva13690-bib-0029] Hiilivirta, P. , Ikonen, E. , & Lappalainen, J. (1998). Comparison of two methods for distinguishing wild from hatchery‐reared salmon (*Salmo salar* Linnaeus, 1758) in the Baltic Sea. ICES Journal of Marine Science, 55(6), 981–986. 10.1006/jmsc.1998.0370

[eva13690-bib-0030] Hoelzel, A. R. , Bruford, M. W. , & Fleischer, R. C. (2019). Conservation of adaptive potential and functional diversity. Conservation Genetics, 20(1), 1–5. 10.1007/s10592-019-01151-x

[eva13690-bib-0031] Huusko, A. , & Hyvärinen, P. (2012). Atlantic salmon abundance and size track climate regimes in the Baltic Sea. Boreal Environment Research, 17(2), 139–149.

[eva13690-bib-0032] ICES . (2011). *Report of the workshop on age determination of Salmon (WKADS). ICES CM/ACOM:44*, (January), 18–20.

[eva13690-bib-0033] ICES . (2023). Baltic Salmon and Trout assessment working group (WGBAST). ICES Scientific Reports, 5(53), 465. 10.17895/ices.pub.4979

[eva13690-bib-0034] Jacobson, P. , Gårdmark, A. , & Huss, M. (2020). Population and size‐specific distribution of Atlantic salmon *Salmo salar* in the Baltic Sea over five decades. Journal of Fish Biology, 96(2), 408–417. 10.1111/jfb.14213 31755101 PMC7028083

[eva13690-bib-0035] Järvi, T. H. (1938). *Fluctuations in the Baltic stock of salmon*. Rapp. PV. Réun. Cons. Int. Explor. Mer, 106.

[eva13690-bib-0036] Jensen, A. J. , Hagen, I. J. , Czorlich, Y. , Bolstad, G. H. , Bremset, G. , Finstad, B. , Hindar, K. , Skaala, Ø. , & Karlsson, S. (2022). Large‐effect loci mediate rapid adaptation of salmon body size after river regulation. Proceedings of the National Academy of Sciences of the United States of America, 119(44), 1–8. 10.1073/pnas.2207634119 PMC9636922 36279467

[eva13690-bib-0037] Jokikokko, E. , Kallio‐Nyberg, I. , & Jutila, E. (2004). The timing, sex and age composition of the wild and reared Atlantic salmon ascending the Simojoki River, northern Finland. Journal of Applied Ichthyology, 20(1), 37–42. 10.1111/j.1439-0426.2004.00491.x

[eva13690-bib-0078] Jonsson, B. , & Jonsson, N. (2011). Ecology of Atlantic Salmon and Brown Trout—Habitat as a template for life histories. Fish and Fisheries Series (Vol. 33). Springer.

[eva13690-bib-0038] Jørgensen, C. , Enberg, K. , Dunlop, E. S. , Arlinghaus, R. , Boukal, D. S. , Brander, K. , Ernande, B. , Gardmark, A. , Johnston, F. , Matsumura, S. , Pardoe, H. , Raab, K. , Silva, A. , Vainikka, A. , Dieckmann, U. , Heino, M. , & Rijnsdorp, A. D. (2007). Ecology: Managing evolving fish stocks. Science, 318(5854), 1247–1248. 10.1126/science.1148089 18033868

[eva13690-bib-0039] Kallio‐Nyberg, I. , Koljonen, M.‐L. , & Saloniemi, I. (2014). Spawning‐age differences and their temporal trends in wild and sea‐ranched Atlantic Salmon stocks, from stock mixture data. The Open Fish Science Journal, 7(1), 46–58. 10.2174/1874401x01407010046

[eva13690-bib-0040] Karlsson, L. , & Karlström, Ö. (1994). The Baltic salmon (*Salmo salar* L.): Its history, present situation and future. Dana, 10, 61–85.

[eva13690-bib-0041] Koljonen, M.‐L. (2006). Annual changes in the proportions of wild and hatchery Atlantic salmon (*Salmo salar*) caught in the Baltic Sea. ICES Journal of Marine Science, 63(7), 1274–1285. 10.1016/j.icesjms.2006.04.010

[eva13690-bib-0042] Koljonen, M.‐L. , & McKinnell, S. (1996). Assessing seasonal changes in stock composition of Atlantic salmon catches in the Baltic Sea with genetic stock identification. Journal of Fish Biology, 49(5), 998–1018. 10.1006/jfbi.1996.0228

[eva13690-bib-0043] Kovach, R. P. , Ellison, S. C. , Pyare, S. , & Tallmon, D. A. (2015). Temporal patterns in adult salmon migration timing across southeast Alaska. Global Change Biology, 21(5), 1821–1833. 10.1111/gcb.12829 25482609

[eva13690-bib-0044] Kurland, S. , Ryman, N. , Hössjer, O. , & Laikre, L. (2023). Effects of subpopulation extinction on effective size (Ne) of metapopulations. Conservation Genetics, 24(4), 417–433. 10.1007/s10592-023-01510-9

[eva13690-bib-0045] Miettinen, A. , Palm, S. , Dannewitz, J. , Lind, E. , Primmer, C. R. , Romakkaniemi, A. , Östergren, J. , & Pritchard, V. L. (2021). A large wild salmon stock shows genetic and life history differentiation within, but not between, rivers. Conservation Genetics, 22(1), 35–51. 10.1007/s10592-020-01317-y

[eva13690-bib-0046] Mobley, K. B. , Aykanat, T. , Czorlich, Y. , House, A. , Kurko, J. , Miettinen, A. , Moustakas‐Verho, J. , Salgado, A. , Sinclair‐Waters, M. , Verta, J.‐P. , & Primmer, C. R. (2021). Maturation in Atlantic salmon (*Salmo salar*, Salmonidae): A synthesis of ecological, genetic, and molecular processes. Reviews in Fish Biology and Fisheries, 31, 523–571. 10.1007/s11160-021-09656-w

[eva13690-bib-0047] Moran, B. M. , & Anderson, E. C. (2019). Bayesian inference from the conditional genetic stock identification model. Canadian Journal of Fisheries and Aquatic Sciences, 76(4), 551–560. 10.1139/cjfas-2018-0016

[eva13690-bib-0048] Morita, K. (2019). Earlier migration timing of salmonids: An adaptation to climate change or maladaptation to the fishery? Canadian Journal of Fisheries and Aquatic Sciences, 76(3), 475–479. 10.1139/cjfas-2018-0078

[eva13690-bib-0049] Myrvold, K. M. , Mawle, G. W. , & Aas, Ø. (2019). 1668 The Social, Economic and Cultural values of wild Atlantic salmon sessment of changes in values.

[eva13690-bib-0050] Niemelä, E. , Orell, P. , Erkinaro, J. , Dempson, J. B. , BrØrs, S. , Svenning, M. A. , & Hassinen, E. (2006). Previously spawned Atlantic salmon ascend a large subarctic river earlier than their maiden counterparts. Journal of Fish Biology, 69(4), 1151–1163. 10.1111/j.1095-8649.2006.01190.x

[eva13690-bib-0051] Östergren, J. , Palm, S. , Gilbey, J. , Spong, G. , Dannewitz, J. , Königsson, H. , Persson, J. , & Vasemägi, A. (2021). A century of genetic homogenization in Baltic salmon – Evidence from archival DNA. Proceedings of the Royal Society B: Biological Sciences, 288, 20203147. 10.1098/rspb.2020.3147 PMC8059615 33878928

[eva13690-bib-0052] Pakarinen, T. , Romakkaniemi, A. , & Leinonen, T. (2022). Pohjanlahden rannikon lohenkalastuksen säätelyn muutokset 2017 ja sen vaikutuksia vuosina 2017–2021: Väliraportti. Luonnonvara‐ ja biotalouden tutkimus, 63, 61s.

[eva13690-bib-0053] Palm, S. , Romakkaniemi, A. , Dannewitz, J. , Pakarinen, T. , Veneranta, L. , Huusko, R. , Isometsä, K. , Broman, A. , & Miettinen, A. (2023). Torneälvens bestånd av lax, havsöring, vandringssik och harr – gemensamt svensk‐finskt biologiskt underlag för bedömning av lämpliga fiskeregler under 2023, 1(52), 1–52.

[eva13690-bib-0054] Pohja‐Mykrä, M. , Matilainen, A. , Kujala, S. , Hakala, O. , Harvio, V. , Törmä, H. , & Kurki, S. (2018). Erätalouteen liittyvän yritystoiminnan nykytila ja kehittämisedellytykset.

[eva13690-bib-0055] Pokki, H. , Artell, J. , Mikkola, J. , Orell, P. , & Ovaskainen, V. (2018). Valuing recreational salmon fishing at a remote site in Finland: A travel cost analysis. Fisheries Research, 208(July), 145–156. 10.1016/j.fishres.2018.07.013

[eva13690-bib-0056] Pritchard, V. L. , Mäkinen, H. , Vähä, J.‐P. , Erkinaro, J. , Orell, P. , & Primmer, C. R. (2018). Genomic signatures of fine‐scale local selection in Atlantic salmon suggest involvement of sexual maturation, energy homeostasis and immune defence‐related genes. Molecular Ecology, 27, 2560–2575. 10.1111/mec.14705 29691916

[eva13690-bib-0057] Quinn, T. P. , McGinnity, P. , & Cross, T. F. (2006). Long‐term declines in body size and shifts in run timing of Atlantic salmon in Ireland. Journal of Fish Biology, 68(6), 1713–1730. 10.1111/j.1095-8649.2006.01017.x

[eva13690-bib-0058] R Core Team . (2021). R: A language and environment for statistical computing. R Foundation for Statistical Computing. https://www.R‐project.org/

[eva13690-bib-0059] Romakkaniemi, A. , Perä, I. , Karlsson, L. , Jutila, E. , Carlsson, U. , & Pakarinen, T. (2003). Development of wild Atlantic salmon stocks in the rivers of the northern Baltic Sea in response to management measures. ICES Journal of Marine Science, 60(2), 329–342. 10.1016/S1054-3139(03)00020-1

[eva13690-bib-0060] Säisä, M. , Koljonen, M.‐L. , Gross, R. , Nilsson, J. , Tähtinen, J. , Koskiniemi, J. , & Vasemägi, A. (2005). Population genetic structure and postglacial colonization of Atlantic salmon (*Salmo salar*) in the Baltic Sea area based on microsatellite DNA variation. Canadian Journal of Fisheries and Aquatic Sciences, 62(8), 1887–1904. 10.1139/f05-094

[eva13690-bib-0061] Schindler, D. E. , Hilborn, R. , Chasco, B. , Boatright, C. P. , Quinn, T. P. , Rogers, L. A. , & Webster, M. S. (2010). Population diversity and the portfolio effect in an exploited species. Nature, 465(7298), 609–612. 10.1038/nature09060 20520713

[eva13690-bib-0062] Shearer, W. M. (1990). The Atlantic salmon (*Salmo salar* L.) of the North Esk with particular reference to the relationship between both river and sea age and time of return to home waters. Fisheries Research, 10(1–2), 93–123. 10.1016/0165-7836(90)90017-P

[eva13690-bib-0063] Sinclair‐Waters, M. , Nome, T. , Wang, J. , Lien, S. , Kent, M. P. , Sægrov, H. , Florø‐Larsen, B. , Bolstad, G. H. , Primmer, C. R. , & Barson, N. J. (2022). Dissecting the loci underlying maturation timing in Atlantic salmon using haplotype and multi‐SNP based association methods. Heredity, 129(6), 356–365. 10.1038/s41437-022-00570-w 36357776 PMC9709158

[eva13690-bib-0064] Stewart, D. C. , Smith, G. W. , & Youngson, A. F. (2002). Tributary‐specific variation in timing of return of adult Atlantic salmon (*Salmo salar*) to fresh water has a genetic component. Canadian Journal of Fisheries and Aquatic Sciences, 59(2), 276–281. 10.1139/f02-011

[eva13690-bib-0065] Thompson, T. Q. , Renee Bellinger, M. , O'Rourke, S. M. , Prince, D. J. , Stevenson, A. E. , Rodrigues, A. T. , Sloat, M. R. , Speller, C. F. , Yang, D. Y. , Butler, V. L. , Banks, M. A. , & Miller, M. R. (2019). Anthropogenic habitat alteration leads to rapid loss of adaptive variation and restoration potential in wild salmon populations. Proceedings of the National Academy of Sciences of the United States of America, 116(1), 177–186. 10.1073/pnas.1811559115 30514813 PMC6320526

[eva13690-bib-0066] Tillotson, M. D. , & Quinn, T. P. (2018). Selection on the timing of migration and breeding: A neglected aspect of fishing‐induced evolution and trait change. Fish and Fisheries, 19(1), 170–181. 10.1111/faf.12248

[eva13690-bib-0067] Uusi‐Heikkilä, S. , Whiteley, A. R. , Kuparinen, A. , Matsumura, S. , Venturelli, P. A. , Wolter, C. , Slate, J. , Primmer, C. R. , Meinelt, T. , Killen, S. S. , Bierbach, D. , Polverino, G. , Ludwig, A. , & Arlinghaus, R. (2015). The evolutionary legacy of size‐selective harvesting extends from genes to populations. Evolutionary Applications, 8(6), 597–620. 10.1111/eva.12268 26136825 PMC4479515

[eva13690-bib-0068] Vähä, J.‐P. , Erkinaro, J. , Niemelä, E. , & Primmer, C. R. (2007). Life‐history and habitat features influence the within‐river genetic structure of Atlantic salmon. Molecular Ecology, 16, 2638–2654. 10.1111/j.1365-294X.2007.03329.x 17594436

[eva13690-bib-0069] Vähä, J. P. , Erkinaro, J. , Niemelä, E. , Primmer, C. R. , Saloniemi, I. , Johansen, M. , Svenning, M. , & Brørs, S. (2011). Temporally stable population‐specific differences in run timing of one‐sea‐winter Atlantic salmon returning to a large river system. Evolutionary Applications, 4, 39–53. 10.1111/j.1752-4571.2010.00131.x 25567952 PMC3352515

[eva13690-bib-0070] Venables, W. N. , & Ripley, B. D. (2002). Modern applied statistics with S (4th ed.). Springer. 10.1046/j.1467-9884.2003.t01-19-00383_22.x

[eva13690-bib-0071] Waples, R. S. , Ford, M. J. , Nichols, K. , Kardos, M. , Myers, J. , Thompson, T. Q. , Anderson, E. C. , Koch, I. J. , McKinney, G. , Miller, M. R. , Naish, K. , Narum, S. R. , O'Malley, K. G. , Pearse, D. E. , Pess, G. R. , Quinn, T. P. , Seamons, T. R. , Spidle, A. , Warheit, K. I. , & Willis, S. C. (2022). Implications of large‐effect loci for conservation: A review and case study with Pacific Salmon. Journal of Heredity, 113(2), 121–144. 10.1093/jhered/esab069 35575083

[eva13690-bib-0072] Whitlock, M. C. , & Lotterhos, K. E. (2015). Reliable detection of loci responsible for local adaptation: Inference of a null model through trimming the distribution of FST. American Naturalist, 186(october), S24–S36. 10.1086/682949 26656214

[eva13690-bib-0073] Whitlock, R. , Mäntyniemi, S. , Palm, S. , Dannewitz, J. , & Östergren, J. (2018). Integrating genetic analysis of mixed populations with a spatially explicit population dynamics model. Methods in Ecology and Evolution, 2018, 1017–1035. 10.1111/2041-210X.12946

[eva13690-bib-0074] Whitlock, R. , Pakarinen, T. , Palm, S. , Koljonen, M. L. , Östergren, J. , & Dannewitz, J. (2021). Trade‐offs among spatio‐temporal management actions for a mixed‐stock fishery revealed by Bayesian decision analysis. ICES Journal of Marine Science, 78(10), 3625–3638. 10.1093/icesjms/fsab203

[eva13690-bib-0076] Yano, A. , Nicol, B. , Jouanno, E. , Quillet, E. , Fostier, A. , Guyomard, R. , & Guiguen, Y. (2013). The sexually dimorphic on the Y‐chromosome gene (sdY) is a conserved male‐specific Y‐chromosome sequence in many salmonids. Evolutionary Applications, 6(3), 486–496. 10.1111/eva.12032 23745140 PMC3673476

[eva13690-bib-0077] Zeileis, A. , & Hothorn, T. (2002). Diagnostic checking in regression relationships. R News, 2(3), 7–10.

