# Supplementary material for: Temporal allele frequency changes in large‐effect loci reveal potential fishing impacts on salmon life‐history diversity
Source: Evol Appl. 2024 Apr 25;17(4):e13690. doi: 10.1111/eva.13690 (PMC11046039; doi:10.1111/eva.13690)
Supplement: Supplementary file 1 — Data S1. [file EVA-17-e13690-s001.docx]

**SUPPLEMENTARY MATERIALS**

**Temporal allele frequency changes in large-effect loci reveal potential fishing impacts on salmon life-history diversity**

**Journal:** *Evolutionary Applications*

**Authors:** Antti Miettinen*, Atso Romakkaniemi, Johan Dannewitz, Tapani Pakarinen, Stefan Palm, Lo Persson, Johan Östergren, Craig R. Primmer, Victoria L. Pritchard

**Corresponding author:** Antti Miettinen ([antmiet@gmail.com](mailto:antmiet@gmail.com))

**Contents:** This supplementary file includes **Tables S1-S5**, **Figures S1-S10** and a **Supplementary Methods and Results** section with **Table SX1** and **Figures SX1-SX3**.

**Main supplementary tables**

**Table S1.** Temporal quartiles (Q) used in this study for qualitative exploration of stock composition changes in within-season salmon catches. Median quartile days (MQD) per area and time point were used for visualisation purposes in Figure 3 and Figure S2.

| **Area** | **Years** | **Q1** | **Q2** | **Q3** | **Q4** |
| --- | --- | --- | --- | --- | --- |
| **Coastal areas** | | | | | |
| Merikarvia (C1) | 2019-2020 | 12 May - 26 May, n = 50 | 27 May - 1 June, n = 44 | 2 June - 5 June, n = 46 | 6 June - 17 July, n = 45 |
|  |  | Median quartile day (MQD) as days since May 1 = 20 | MQD = 28 | MQD = 41 | MQD = 55 |
| Luoto (C2) | 2019-2020 | 28 May - 12 June, n = 64 | 13 June - 19 June, n = 65 | 20 June - 1 July, n = 59 | 3 July - 22 July, n = 64 |
|  |  | MQD = 35 | MQD = 47 | MQD = 55 | MQD = 69 |
| Kemi River mouth (C3) | 2019-2020 | 2 June - 14 June, n = 38 | 15 June - 20 June, n = 42 | 21 June - 25 June, n = 52 | 26 June - 3 July, n = 46 |
|  |  | MQD = 41 | MQD = 47 | MQD = 52 | MQD = 60 |
| Tornio River mouth (C4) | 2019-2020 | 11 June - 18 June, n = 83 | 19 June - 23 June, n = 94 | 24 June - 29 June, n = 102 | 30 June - 20 July, n = 81 |
|  |  | MQD = 47 | MQD = 51 | MQD = 56 | MQD = 69 |
| Seskarö and Bergön, Sweden (C5) | 2020 | No quartiles were used for this area due to a small sample size (n = 114) and short fishing season. | | | |
| **Tornio River** | | | | | |
| Downstream (R1) | 2019-2020 | 1 June - 2 June, n = 45 | 3 June - 5 June, n = 35 | 6 June - 15 June, n = 37 | 16 June - 12 Aug., n = 34 |
|  |  | MQD = 32 | MQD = 34 | MQD = 41 | MQD = 53.5 |
| Pello-Lappea (R2) | 2019-2020 | 2 June - 24 June, n = 88 | 25 June - 1 Aug., n = 78 | 2 Aug. - 15 Aug., n = 87 | 16 Aug. - 30 Aug., n = 76 |
|  |  | MQD = 41 | MQD = 65.5 | MQD = 99 | MQD = 113.5 |
| Kihlanki (R3) | 2019-2020 | 4 June - 9 July, n = 53 | 11 July - 5 Aug., n = 53 | 6 Aug. - 20 Aug., n = 55 | 21 Aug. - 31 Aug., n = 55 |
|  |  | MQD = 60 | MQD = 86 | MQD = 106 | MQD = 117 |
| Tornio River combined (R1-R3) | 2014-2016 | 1 June - 13 June, n = 50 | 14 June - 25 June, n = 50 | 26 June - 31 July, n = 55 | 1 Aug. - 31 Aug., n = 53 |
|  |  | MQD = 37 | MQD = 48 | MQD = 60 | MQD = 109 |
| Tornio River combined (R1-R3) | 2004-2006 | 26 May - 18 June, n = 34 | 19 June - 27 June, n = 34 | 28 June - 22 July, n = 32 | 23 July - 15 Aug., n = 37 |
|  |  | MQD = 40 | MQD = 53.5 | MQD = 63 | MQD = 95 |

**Table S2.** Estimated accuracy of the genetic stock identification method (“*self-assignment accuracy*”) to distinguish different origins of individuals in the catches of wild salmon. We used 166 SNP markers and the *rubias* analysis method to assess this.

| **Origin** | **Estimate of identification accuracy (%)** |
| --- | --- |
| Lower Tornio-Kalix | 89.2 |
| Upper Tornio-Kalix | 88.7 |
| Upper Lainio | 95.0 |
| Ängesån | 87.9 |
| Simo River | 96.4 |
| Råne River | 100 |
| Vindel River | 100 |
| Kymi River | 100 |

**Table S3.** Estimated stock proportions in coastal and Tornio River catches of wild Baltic salmon from 2019-2020.

| **Origin** | **Coastal** | | **Tornio River** | |
| --- | --- | --- | --- | --- |
|  | **Proportion (%)** | **95% CI** | **Proportion (%)** | **95% CI** |
| Lower Tornio-Kalix | 75.9 | 73.1 – 78.4 | 80.5 | 77.2 – 83.5 |
| Upper Tornio-Kalix | 17.5 | 15.1 – 20.1 | 10.5 | 8.2 – 13.1 |
| Upper Lainio River | 2.8 | 1.8 – 4 | 8.6 | 6.7 – 10.9 |
| Ängesån | 1.2 | 0.6 – 2.1 | 0.3 | 0 – 0.9 |
| Simo River | 1.3 | 0.7 – 2.1 | 0 | 0 – 0.2 |
| Råne River | 0.2 | 0 – 0.5 | 0 | 0 – 0.2 |
| Vindel River | 0.8 | 0.4 – 1.4 | 0 | 0 – 0.1 |
| Kymi River stock* | 0.2 | 0 – 0.5 | 0 | 0 – 0.2 |

*Also represents other eastern/southeastern Baltic salmon stocks in the catches.

**Tables S4a-d:** Results of the statistical models used.

**Table S4a.** Statistical associations of *vgll3* and *six6* genotypes with age at maturity in the entire Baltic salmon catch dataset from 1928 to 2020.

| **Sex** | **Model** | **lnLK** | **p** |
| --- | --- | --- | --- |
| Males | Full model | -1007.6 |  |
|  | Without *six6* dominance | -1008.1 | 0.61 |
|  | Without *vgll3* dominance | -1024.0 | < 7.3e-08 |
|  | Without *six6* additive | -1020.2 | < 3.7e-06 |
|  | Without *vgll3* additive | -1116.4 | < 2.2e-16 |
| Females | Full model | -1118.8 |  |
|  | Without *six6* dominance | -1119.4 | 0.57 |
|  | Without *vgll3* dominance | -1119.3 | 0.60 |
|  | Without *six6* additive | -1147.3 | < 4.20e-13 |
|  | Without *vgll3* additive | -1134.2 | < 2.21e-07 |


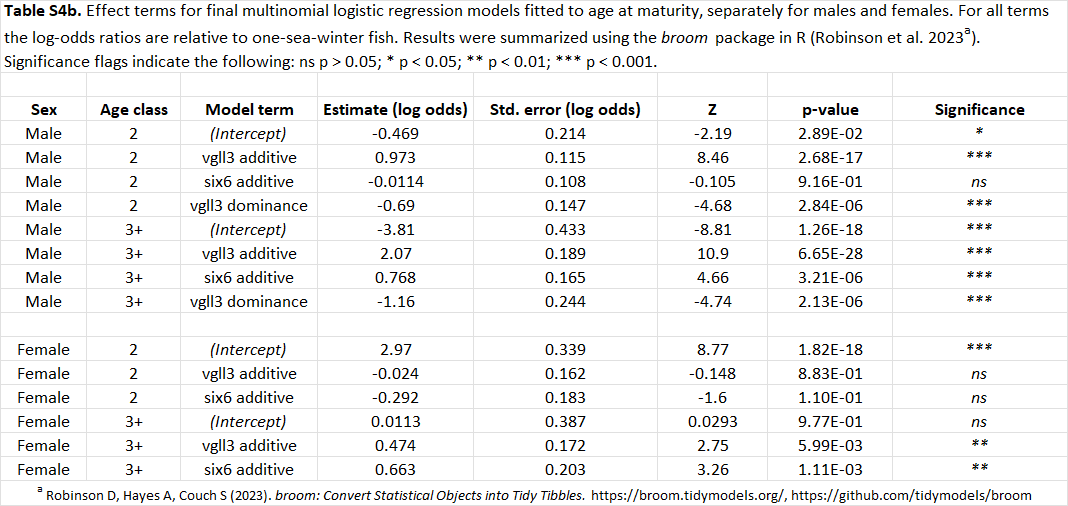


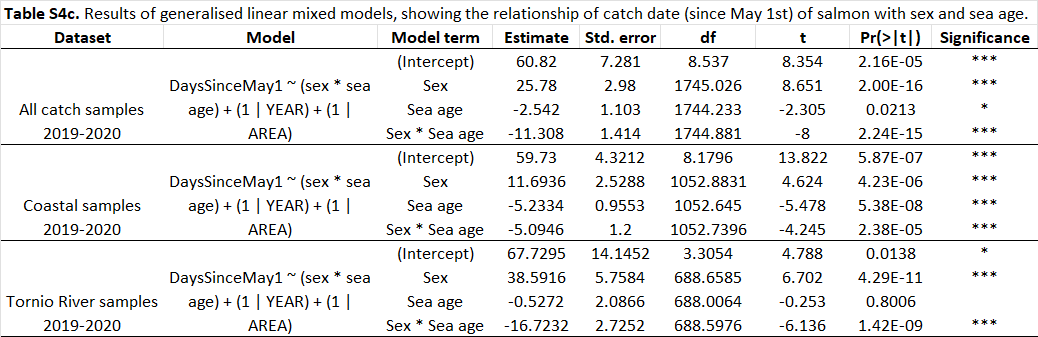


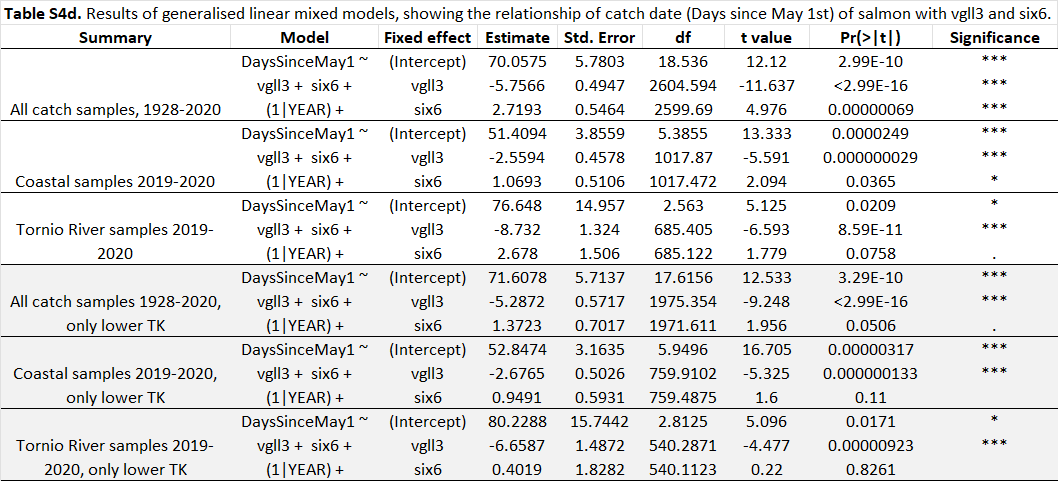


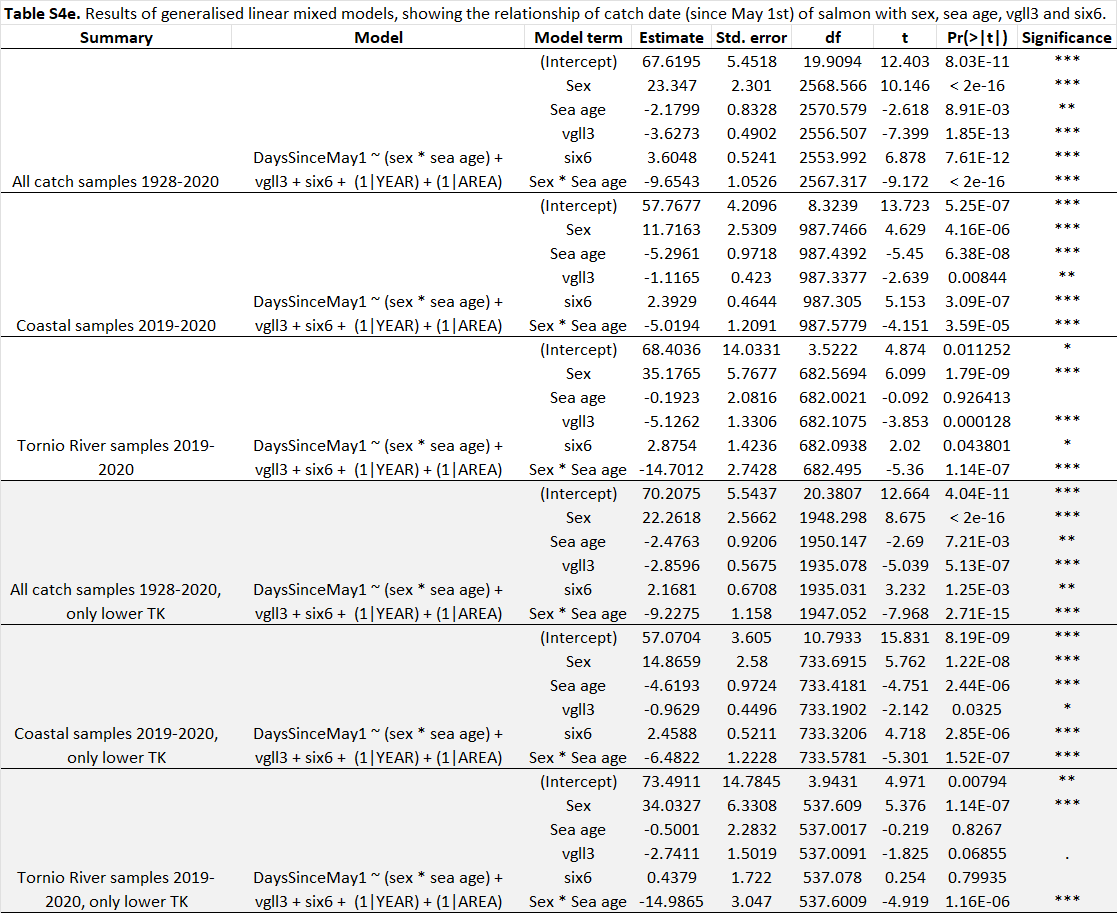


**Table S5.** Information about the 229/169 SNPs used in the GT-seq panel. Chr = chromosome, position refers to the physical position (bp) on each chromosome, and V2 and V3 refer to the Atlantic salmon reference genome assemblies ICSASG_v2 and Ssal_v3.1, respectively. SNP categories: GSI = SNPs chosen based on their power for genetic stock identification (GSI) of Baltic salmon, Swedish = SNPs used in Östergren et al. (2021), Targeted = SNPs linked to genes of possible adaptive significance in Teno/Tana River salmon (Johansson et al., in prep.), Teno = SNPs shared with an existing, validated panel developed for GSI in the Teno/Tana River (Johansson et al., in prep.), TK outlier = SNPs potentially under local selection within the Tornio-Kalix river system (see Supplementary Methods and Results).

| **SNP** | **Chr (V2)** | **Position (V2)** | **Chr (V3)** | **Position (V3)** | **SNP category** | **In final panel** |
| --- | --- | --- | --- | --- | --- | --- |
| AX-96433380 | 1 | 27827340 | 1 | 30652880 | GSI | Yes |
| AX-87017797 | 1 | 39548651 | 1 | 42500770 | GSI | Yes |
| AX-87183638 | 1 | 40967828 | 1 | 43503121 | Teno | Yes |
| AX-96452221 | 1 | 54492238 | 1 | 57296239 | GSI | Yes |
| AX-87058719 | 1 | 62145423 | 1 | 64983239 | GSI | Yes |
| AX-96121687 | 1 | 82977344 | 1 | 97383667 | GSI | Yes |
| AX-87387594 | 1 | 92630439 | 1 | 79028101 | Teno | Yes |
| AX-96466221 | 1 | 110964612 | 1 | 125833582 | GSI | Yes |
| AX-87044662 | 1 | 116526708 | 18 | 37446213 | Teno | Yes |
| AX-87307234 | 1 | 117168938 | 1 | 130677342 | GSI | Yes |
| AX-96466162 | 1 | 129624468 | 1 | 143123861 | GSI | Yes |
| AX-86910809 | 2 | 10277221 | 5 | 72057694 | GSI | Yes |
| AX-87783878 | 2 | 21435207 | 2 | 32631174 | Teno | Yes |
| AX-87082256 | 2 | 48086119 | 2 | 58214156 | Teno | Yes |
| AX-98320146 | 2 | 56008165 | 2 | 64663420 | GSI | Yes |
| AX-87695214 | 2 | 59356442 | 2 | 68912621 | Teno | Yes |
| AX-87249571 | 2 | 67134316 | 2 | 78412462 | Teno | Yes |
| AX-96290763 | 3 | 4161842 | 3 | 5977745 | GSI | Yes |
| AX-87424445 | 3 | 7387408 | 3 | 7122097 | GSI | Yes |
| AX-87868321 | 3 | 21602968 | 3 | 23448086 | GSI | Yes |
| AX-87358187 | 3 | 28973673 | 3 | 30708383 | Teno | Yes |
| AX-215425781 | 3 | 53605546 | 3 | 53756129 | GSI | Yes |
| AX-86999175 | 4 | 34186706 | 4 | 39387246 | GSI | Yes |
| AX-87803701 | 4 | 38930773 | 4 | 44238918 | GSI | Yes |
| AX-87708993 | 4 | 40455173 | 4 | 45796906 | GSI | Yes |
| AX-87212974 | 4 | 49556883 | 4 | 54982636 | Targeted *(btg3)* | Yes |
| AX-96479307 | 4 | 50442470 | 4 | 55921473 | GSI | Yes |
| AX-87099457 | 4 | 51077376 | 4 | 56560283 | GSI | Yes |
| AX-86981356 | 4 | 59914225 | 4 | 65567156 | GSI | Yes |
| AX-87663735 | 5 | 16391077 | 5 | 17471500 | GSI | Yes |
| AX-215411712 | 5 | 28977020 | 5 | 31574408 | GSI | Yes |
| AX-87848834 | 5 | 38753635 | 5 | 40648242 | Teno | Yes |
| AX-87853873 | 5 | 41790709 | 5 | 43701947 | Teno | Yes |
| AX-87644039 | 5 | 51817063 | 5 | 54420560 | GSI | Yes |
| AX-87061895 | 5 | 56987120 | 5 | 59619722 | GSI | Yes |
| AX-87610668 | 5 | 68074662 | 5 | 69860402 | GSI | Yes |
| AX-87749553 | 5 | 68296219 | 5 | 70095722 | GSI | Yes |
| AX-87065107 | 6 | 5674332 | 6 | 9159983 | Swedish | Yes |
| AX-215416301 | 6 | 70737518 | 6 | 75840126 | GSI | Yes |
| AX-87455840 | 7 | 12687870 | 7 | 16035065 | GSI | Yes |
| AX-87239249 | 7 | 16590732 | 7 | 19975727 | GSI | Yes |
| AX-87748163 | 8 | 10319579 | 8 | 10610066 | Teno | Yes |
| AX-87228087 | 9 | 22648087 | 9 | 21585429 | Teno | Yes |
| AX-87434297 | 9 | 24021198 | 9 | 22954424 | Teno | Yes |
| AX-87516950 | 9 | 24407746 | 9 | 23353262 | Teno | Yes |
| AX-87730832 | 9 | 24904601 | 9 | 23855462 | Targeted *(six6_top_)* | Yes |
| AX-87624761 | 9 | 25462494 | 9 | 24431327 | TK outlier | Yes |
| AX-87607030 | 9 | 26044179 | 9 | 25019381 | Teno | Yes |
| AX-86929895 | 9 | 26216585 | 9 | 25185868 | Teno | Yes |
| AX-215438386 | 9 | 29588464 | 9 | 28245816 | GSI | Yes |
| AX-87602770 | 9 | 30337834 | 9 | 29002882 | GSI | Yes |
| AX-87147003 | 9 | 30853386 | 9 | 29566858 | GSI | Yes |
| AX-87615441 | 9 | 36159955 | 9 | 34875360 | GSI | Yes |
| AX-87563285 | 9 | 40810127 | 9 | 39580108 | GSI | Yes |
| AX-87165822 | 9 | 44292576 | 9 | 45593558 | TK outlier | Yes |
| AX-215435575 | 9 | 60294429 | 9 | 66578750 | GSI | Yes |
| AX-96436517 | 9 | 68933366 | 9 | 75695961 | GSI | Yes |
| AX-87559659 | 9 | 73008872 | 9 | 79894902 | Teno | Yes |
| AX-96476158 | 9 | 99104363 | 9 | 114321530 | GSI | Yes |
| AX-87461860 | 9 | 111539563 | 9 | 128231392 | GSI | Yes |
| AX-86953449 | 10 | 5798001 | 10 | 4499237 | Teno | Yes |
| AX-87085015 | 10 | 14201416 | 10 | 16082531 | GSI | Yes |
| AX-215425362 | 10 | 29186889 | 10 | 31146901 | GSI | Yes |
| AX-87612930 | 10 | 66175975 | 10 | 73845406 | Teno | Yes |
| AX-87528509 | 10 | 92123334 | 10 | 101469514 | Teno | Yes |
| AX-87318648 | 10 | 93558752 | 10 | 104668347 | Teno | Yes |
| AX-87607648 | 10 | 105345167 | 10 | 114100707 | GSI | Yes |
| AX-87365248 | 11 | 12418225 | 11 | 12445753 | GSI | Yes |
| AX-87283593 | 11 | 19205606 | 11 | 19276289 | Targeted *(numa1)* | Yes |
| AX-215406373 | 11 | 41781912 | 11 | 37998743 | GSI | Yes |
| AX-87801180 | 11 | 48333444 | 11 | 63116602 | Teno | Yes |
| AX-96199591 | 11 | 49727578 | 11 | 64576822 | GSI | Yes |
| AX-87734987 | 11 | 63333952 | 11 | 78271418 | GSI | Yes |
| AX-87827382 | 11 | 75755394 | 11 | 90205372 | GSI | Yes |
| AX-87617699 | 12 | 36043309 | 12 | 43581881 | Teno | Yes |
| AX-87536258 | 12 | 61391928 | 12 | 69203713 | Teno | Yes |
| AX-96429301 | 12 | 61602039 | 12 | 69384073 | Targeted *(mhcII)* | Yes |
| AX-86998181 | 13 | 15070311 | 13 | 20203510 | GSI | Yes |
| AX-87833148 | 13 | 15565501 | 13 | 19658836 | GSI | Yes |
| AX-87510248 | 13 | 22574459 | 13 | 33113373 | GSI | Yes |
| AX-87283122 | 13 | 34100084 | 13 | 42899743 | GSI | Yes |
| AX-96137726 | 13 | 42041998 | 13 | 34864151 | GSI | Yes |
| AX-87631946 | 13 | 42377175 | 13 | 34522785 | GSI | Yes |
| AX-87180056 | 13 | 42971026 | 13 | 33918099 | TK outlier | Yes |
| AX-87735778 | 13 | 68991115 | 13 | 73682573 | GSI | Yes |
| AX-87058808 | 13 | 72459514 | 13 | 77105796 | Teno | Yes |
| AX-87493254 | 13 | 73614288 | 13 | 78281516 | GSI | Yes |
| AX-86957252 | 13 | 84096358 | 13 | 88683388 | GSI | Yes |
| AX-87457194 | 14 | 13085539 | 14 | 13203694 | Teno | Yes |
| AX-87325526 | 14 | 23960261 | 14 | 24305349 | TK outlier | Yes |
| AX-87455641 | 14 | 31171021 | 14 | 31590673 | Teno | Yes |
| AX-87077459 | 14 | 58096830 | 14 | 63383961 | GSI | Yes |
| AX-87647989 | 14 | 66681360 | 14 | 72572120 | GSI | Yes |
| AX-87028170 | 14 | 73740393 | 14 | 80283980 | Teno | Yes |
| AX-86996993 | 14 | 74928868 | 14 | 81511720 | GSI | Yes |
| AX-87023269 | 14 | 89439292 | 14 | 95718840 | Teno | Yes |
| AX-86975012 | 15 | 30067612 | 15 | 35464213 | Teno | Yes |
| AX-87319844 | 15 | 85014715 | 15 | 89748626 | Teno | Yes |
| AX-96441097 | 16 | 33944303 | 16 | 34560035 | TK outlier | Yes |
| AX-87058313 | 16 | 40540241 | 16 | 41190204 | Teno | Yes |
| AX-86998768 | 16 | 40942245 | 16 | 55155843 | GSI | Yes |
| AX-86929052 | 16 | 62594771 | 16 | 63991430 | GSI | Yes |
| AX-87000402 | 16 | 76636610 | 16 | 75390125 | Teno | Yes |
| AX-87795424 | 17 | 4855979 | 17 | 9942194 | Teno | Yes |
| AX-87314393 | 17 | 5802556 | 17 | 11005636 | GSI | Yes |
| AX-87561029 | 17 | 14026819 | 17 | 18544831 | Teno | Yes |
| AX-87383947 | 17 | 20400916 | 17 | 24666586 | Teno | Yes |
| AX-87310045 | 17 | 37624970 | 17 | 54270289 | Teno | Yes |
| AX-96145219 | 17 | 50850961 | 17 | 67344671 | GSI | Yes |
| AX-87263275 | 18 | 6120409 | 18 | 7407441 | GSI | Yes |
| AX-86951802 | 18 | 9477248 | 18 | 10750106 | Teno | Yes |
| AX-88025516 | 18 | 11345036 | 18 | 12621750 | GSI | Yes |
| AX-96299933 | 18 | 43348099 | 18 | 53581286 | GSI | Yes |
| AX-96309731 | 18 | 44647121 | 18 | 54885639 | GSI | Yes |
| AX-87237740 | 18 | 50730902 | 18 | 61002771 | GSI | Yes |
| AX-88264131 | 18 | 56647684 | 18 | 67174612 | GSI | Yes |
| AX-215437481 | 18 | 57911939 | 18 | 68489434 | GSI | Yes |
| AX-87899265 | 18 | 59200892 | 18 | 69546989 | GSI | Yes |
| AX-87482742 | 19 | 12209542 | 19 | 11927172 | GSI | Yes |
| AX-87428769 | 19 | 23223522 | 19 | 23028949 | Teno | Yes |
| AX-87786391 | 19 | 23399990 | 19 | 23217526 | Targeted *(mc4r-like)* | Yes |
| AX-87013456 | 19 | 34669101 | 19 | 40008230 | GSI | Yes |
| AX-87619971 | 19 | 50210579 | 19 | 53980577 | GSI | Yes |
| AX-88174765 | 20 | 8499443 | 20 | 9546027 | GSI | Yes |
| AX-96180231 | 20 | 26466124 | 20 | 27718698 | TK outlier | Yes |
| AX-87543761 | 20 | 39620655 | 20 | 41314299 | GSI | Yes |
| AX-87261797 | 20 | 40620641 | 20 | 42356827 | GSI | Yes |
| AX-87638132 | 20 | 48461198 | 20 | 54146868 | Teno | Yes |
| AX-96126060 | 20 | 53540279 | 20 | 59447981 | GSI | Yes |
| AX-87252671 | 20 | 58431802 | 20 | 64467765 | Teno | Yes |
| AX-87746670 | 20 | 74367600 | 20 | 83864061 | Teno | Yes |
| AX-86947973 | 20 | 79599537 | 20 | 89817889 | GSI | Yes |
| AX-96489623 | 20 | 82239002 | 20 | 91513446 | GSI | Yes |
| AX-87436422 | 21 | 11282511 | 21 | 11340176 | GSI | Yes |
| AX-87340898 | 21 | 31015275 | 21 | 30940598 | GSI | Yes |
| AX-97896532 | 21 | 44199741 | 21 | 43497926 | GSI | Yes |
| AX-87194685 | 21 | 48592209 | 21 | 48071239 | GSI | Yes |
| AX-87428656 | 21 | 48881143 | 21 | 48360100 | GSI | Yes |
| AX-87553012 | 22 | 28798239 | 22 | 29546550 | GSI | Yes |
| AX-87210398 | 22 | 39132396 | 22 | 39585346 | GSI | Yes |
| AX-87359875 | 22 | 62344180 | 22 | 62803965 | GSI | Yes |
| AX-87605253 | 23 | 2357288 | 23 | 2702071 | Teno | Yes |
| AX-87119036 | 23 | 14978152 | 23 | 15424744 | GSI | Yes |
| AX-87443107 | 23 | 21305815 | 23 | 21644011 | Teno | Yes |
| AX-96453267 | 24 | 23912524 | 24 | 24259529 | GSI | Yes |
| AX-87229056 | 24 | 33582135 | 24 | 32254162 | GSI | Yes |
| AX-96416188 | 24 | 36964262 | 24 | 35659347 | GSI | Yes |
| AX-87165669 | 24 | 38373462 | 24 | 38173747 | Swedish | Yes |
| AX-87229293 | 24 | 42564606 | 24 | 39245027 | Teno | Yes |
| AX-96143861 | 25 | 8440040 | 25 | 8786956 | GSI | Yes |
| AX-87465798 | 25 | 13004570 | 25 | 13340106 | Teno | Yes |
| AX-87025000 | 25 | 15635286 | 25 | 15984607 | GSI | Yes |
| AX-215443098 | 25 | 20122393 | 25 | 20330549 | GSI | Yes |
| AX-87420691 | 25 | 28666898 | 25 | 29007416 | Targeted *(vgll3_top_)* | Yes |
| AX-96181276 | 25 | 33519165 | 25 | 33755549 | GSI | Yes |
| AX-87057338 | 26 | 20092707 | 26 | 19971655 | Teno | Yes |
| AX-87776791 | 26 | 24818512 | 26 | 24150344 | Teno | Yes |
| AX-87726184 | 27 | 9851578 | 27 | 10240817 | Teno | Yes |
| AX-87649300 | 27 | 13896277 | 27 | 13213177 | Swedish | Yes |
| AX-87431057 | 27 | 17847203 | 27 | 18283698 | TK outlier | Yes |
| AX-86916436 | 27 | 26383450 | 27 | 26909693 | GSI | Yes |
| AX-87810786 | 27 | 30761557 | 27 | 31332979 | GSI | Yes |
| AX-86984017 | 27 | 39383361 | 27 | 40395607 | GSI | Yes |
| AX-87848427 | 28 | 12780238 | 28 | 12991487 | Swedish | Yes |
| AX-96457574 | 28 | 18801538 | 28 | 19189983 | GSI | Yes |
| AX-87215359 | 28 | 33766342 | 28 | 35440353 | GSI | Yes |
| AX-87809559 | 29 | 7784370 | 29 | 7523931 | GSI | Yes |
| AX-96180604 | 29 | 26711356 | 29 | 26679094 | GSI | Yes |
| sdY marker |  |  |  |  | *sdY* marker | Yes |
| AX-87006342 | 1 | 116770138 | NW_025550921.1 | 17019 | GSI | No |
| AX-87072095 | 1 | 123094944 | 1 | 136637751 | Teno | No |
| AX-96183648 | 1 | 128851360 | 1 | 142355105 | GSI | No |
| AX-86995421 | 1 | 131922560 | 9 | 104327308 | Teno | No |
| AX-215419213 | 1 | 133204703 | 1 | 146900377 | GSI | No |
| AX-215432808 | 2 | 46764373 | 2 | 56901048 | GSI | No |
| AX-87262597 | 3 | 60136721 | 3 | 60524656 | GSI | No |
| AX-87866232 | 3 | 86297956 | 3 | 76317720 | Teno | No |
| AX-87028926 | 4 | 57518969 | 4 | 63142289 | Teno | No |
| AX-87119559 | 4 | 68605017 | 4 | 73721232 | Teno | No |
| AX-87247200 | 4 | 69164294 | 4 | 74357505 | Teno | No |
| AX-87511826 | 5 | 39942372 | 5 | 41842836 | Targeted *(htr4)* | No |
| AX-87284146 | 5 | 45330860 | 5 | 47909324 | Teno | No |
| AX-87573573 | 5 | 46537317 | 5 | 49116826 | GSI | No |
| AX-96476789 | 6 | 29639067 | 6 | 33043396 | GSI | No |
| AX-215437421 | 6 | 84026543 | 6 | 91205211 | GSI | No |
| AX-87182501 | 7 | 12077373 | 7 | 15419362 | Teno | No |
| AX-87048810 | 7 | 26209533 | 7 | 50853740 | GSI | No |
| AX-87425393 | 7 | 33515540 | 7 | 37266244 | GSI | No |
| AX-87472254 | 7 | 42816243 | 7 | 46448500 | Teno | No |
| AX-87103426 | 8 | 14857715 | 4 | 14413070 | Teno | No |
| AX-87541276 | 9 | 26399064 | 9 | 25372318 | Teno | No |
| AX-87230854 | 9 | 27188001 | 9 | 26229357 | TK outlier | No |
| AX-87552531 | 9 | 31608172 | 9 | 30261557 | GSI | No |
| AX-87510610 | 9 | 41512497 | 9 | 40451325 | GSI | No |
| AX-87567766 | 9 | 43830108 | 9 | 44042833 | TK outlier | No |
| AX-87012148 | 9 | 55652349 | 9 | 61950073 | GSI | No |
| AX-86952790 | 9 | 95919822 | 9 | 111116012 | Teno | No |
| AX-86971773 | 10 | 93839605 | 10 | 102853962 | GSI | No |
| AX-215436544 | 10 | 108187444 | 10 | 117070021 | GSI | No |
| AX-215400830 | 11 | 1288920 | 11 | 1219809 | GSI | No |
| AX-87654739 | 11 | 10407528 | 11 | 10441075 | GSI | No |
| AX-96185017 | 11 | 11754020 | 11 | 11780224 | GSI | No |
| AX-87386696 | 12 | 7736453 | 12 | 14898291 | Teno | No |
| AX-87758687 | 12 | 61599355 | 12 | 69381916 | Targeted *(mhcII)* | No |
| AX-215414905 | 13 | 47647941 | 13 | 52295051 | GSI | No |
| AX-87664535 | 13 | 66198952 | 13 | 70973625 | Teno | No |
| AX-87691984 | 13 | 81495521 | 13 | 86195976 | GSI | No |
| AX-87223810 | 14 | 3720637 | 14 | 3862534 | GSI | No |
| AX-96147371 | 14 | 10362571 | 14 | 10487893 | GSI | No |
| AX-88282579 | 14 | 12455032 | 14 | 12573278 | GSI | No |
| AX-96504037 | 14 | 17729775 | 14 | 17917933 | GSI | No |
| AX-96157574 | 14 | 36544414 | 14 | 36614803 | Swedish | No |
| AX-87729953 | 14 | 52731217 | 14 | 57942481 | GSI | No |
| AX-87178879 | 15 | 87061866 | 15 | 91668883 | GSI | No |
| AX-87110072 | 16 | 2185015 | 16 | 2781550 | Teno | No |
| AX-87006420 | 17 | 48592713 | 17 | 65837436 | GSI | No |
| AX-87374958 | 18 | 57872537 | 18 | 68469418 | GSI | No |
| AX-87184558 | 19 | 66865221 | 19 | 70009451 | Swedish | No |
| AX-87035276 | 20 | 6014231 | 20 | 7054272 | TK outlier | No |
| AX-87033641 | 21 | 30174575 | 21 | 30096976 | GSI | No |
| AX-96135192 | 21 | 47888798 | 21 | 47325505 | GSI | No |
| AX-87517899 | 21 | 48298644 | 21 | 47783173 | GSI | No |
| AX-87637642 | 21 | 49911444 | NW_025549003.1 | 14498 | TK outlier | No |
| AX-87312064 | 22 | 13693074 | 22 | 14268508 | GSI | No |
| AX-87104909 | 22 | 56840384 | 22 | 55113774 | GSI | No |
| AX-87140820 | 24 | 22233811 | 24 | 22584704 | GSI | No |
| AX-87776217 | 25 | 21032881 | 25 | 21378445 | Teno | No |
| AX-87083917 | 25 | 28720779 | 25 | 29064238 | Targeted *(akap11)* | No |
| AX-87199935 | 25 | 44073291 | 25 | 44765577 | Teno | No |

**Main supplementary figures**


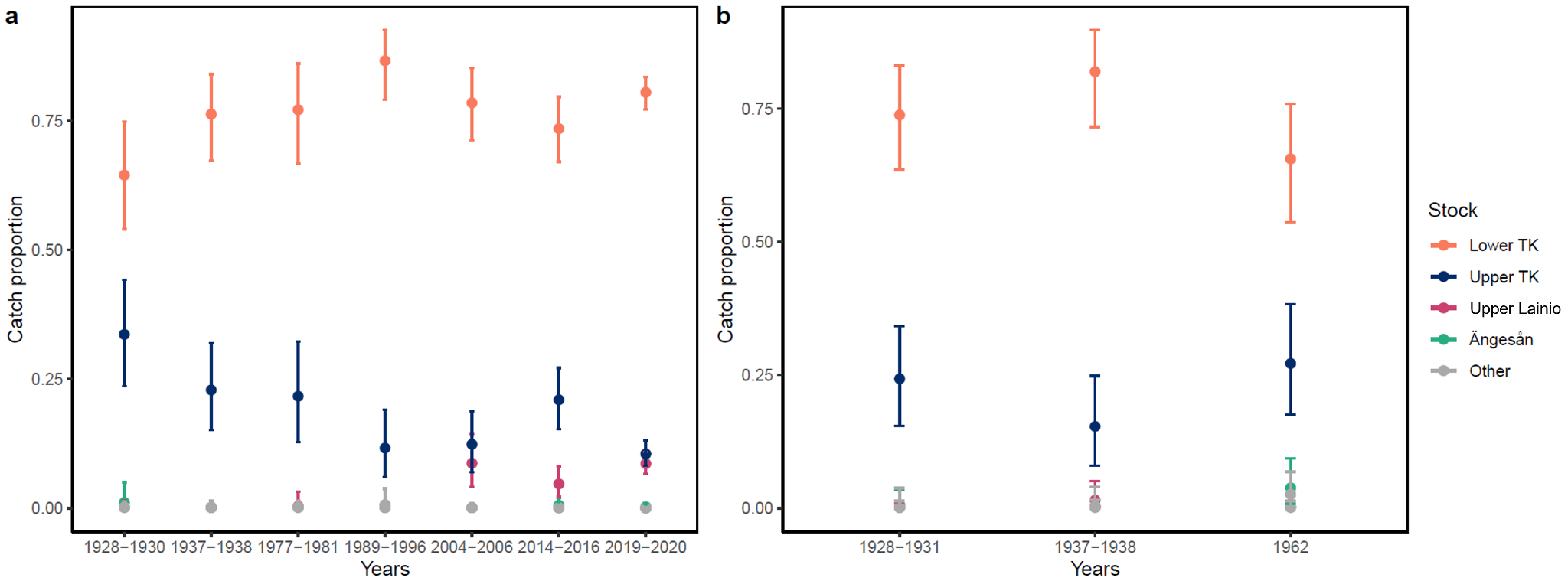


**Figure S1.** Estimated stock proportions in **a** Tornio River salmon catches from 1928 to 2020 and **b** Kalix River salmon catches from 1928 to 1962. The error bars denote 95% credible intervals. TK refers to Tornio-Kalix.


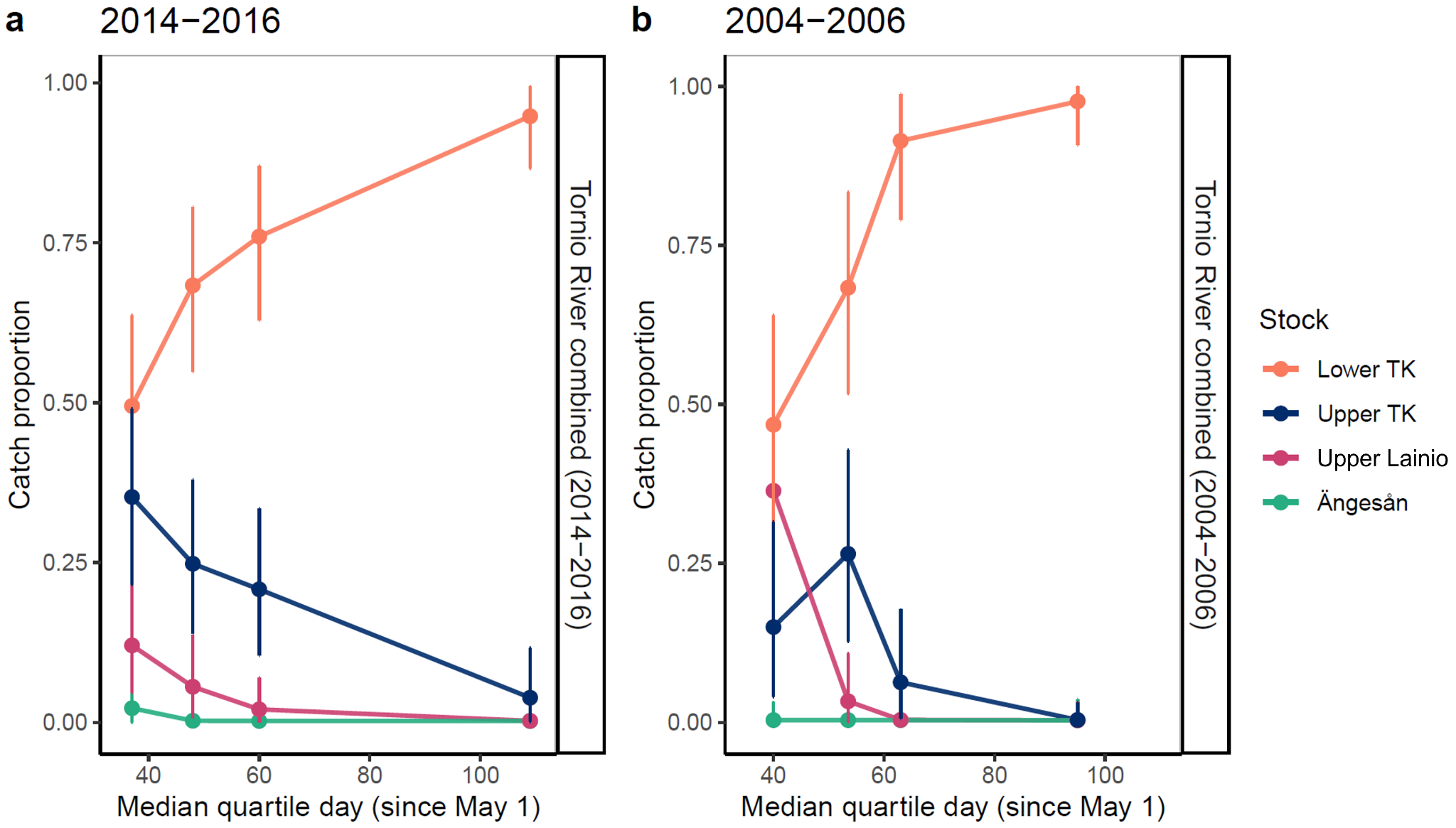


**Figure S2.** Estimated stock proportions in Tornio River salmon catches over the seasons, collected during **a** 2014-2016 and **b** 2004-2006. The catches were split temporally into quartiles based on the number of genetic samples. The median quartile day refers to the median catch date of individuals in each quartile, counted as days since May 1 (see Table S1). The error bars denote 95% credible intervals. TK refers to Tornio-Kalix.


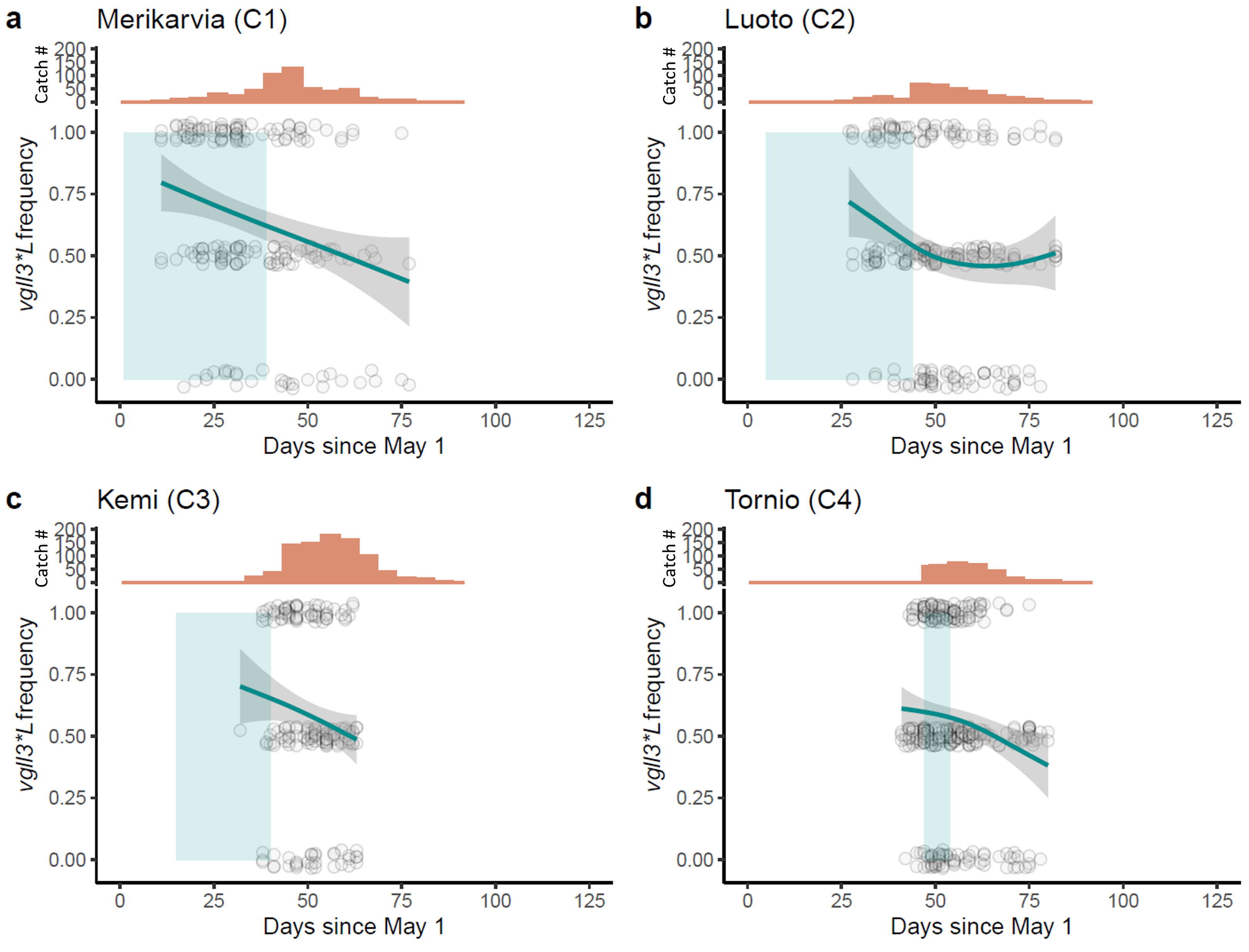


**Figure S3.** Frequency of *vgll3*L* (associated with older age at maturity) in wild coastal salmon catches over the fishing season in **a** C1**/**Merikarvia, **b** C2/Luoto, **c** C3/Kemi River mouth, and **d** C4**/**Tornio River mouth fishing areas during 2019-2020. The histograms show the estimated daily catch sizes (number of salmon caught per day) in these areas for the duration of the entire fishing season (May to July). The line depicts a relationship between *vgll3*L* and catch date, fitted with a GAM, whereas the grey area around the line illustrates the uncertainty of the fitted relationship. The data points represent the *vgll3* genotype of individual samples. The points are jittered on the y-axis to aid figure interpretation. The shaded rectangles represent the time span during the season when fishing was not allowed prior to 2017 (“advanced fishing season”). Note that legal fishing seasons are different for different fishing areas. TK refers to Tornio-Kalix.


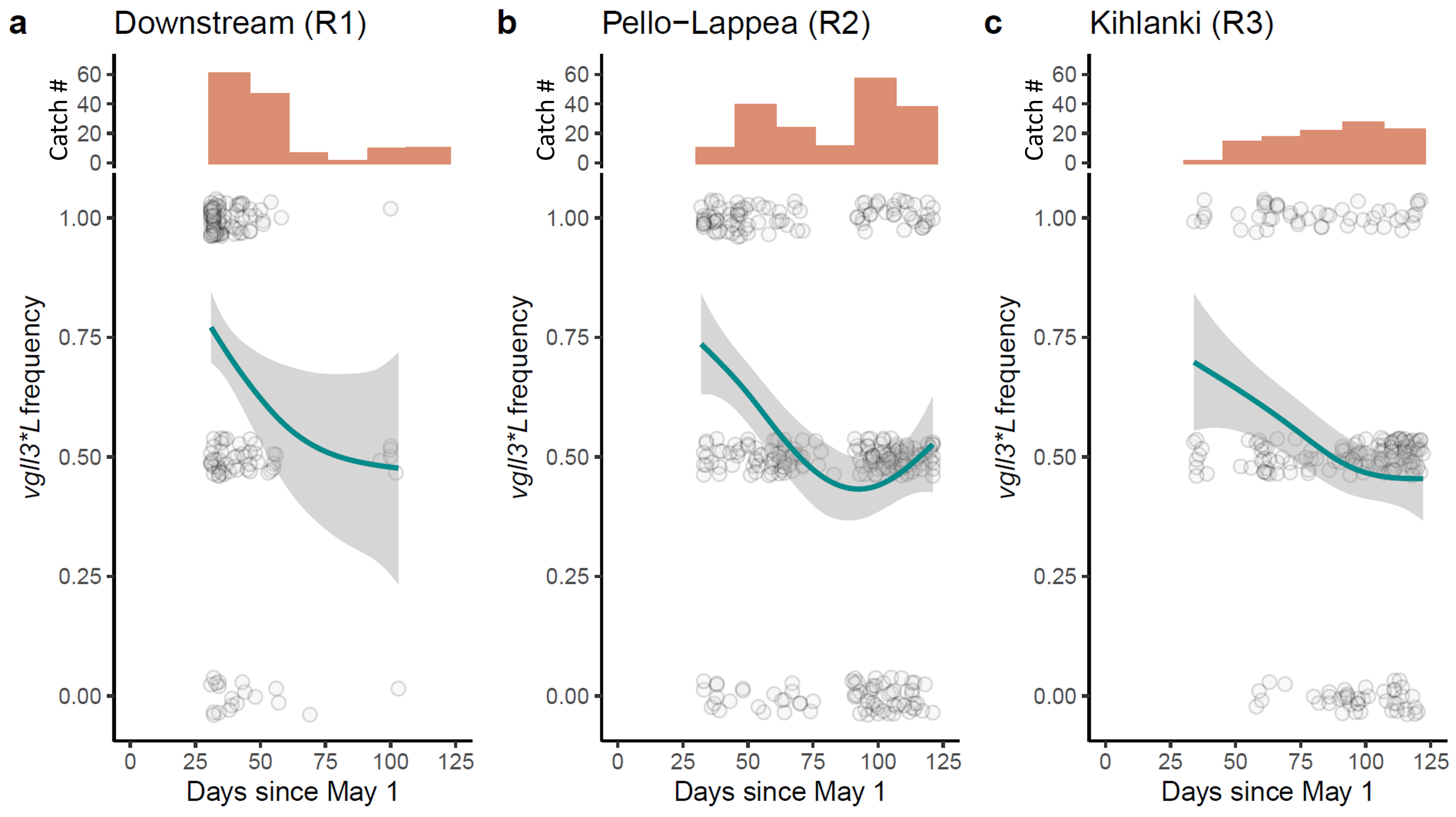


**Figure S4.** Frequency of *vgll3*L* (associated with older age at maturity) in wild salmon catches from the Tornio River over the fishing season in **a** the Downstream, **b** Pello-Lappea, and **c** Kihlanki fishing areas during 2019-2020. The histograms show the estimated daily catch sizes (number of salmon caught per day) in these areas for the duration of the fishing season (from June to August). The line depicts a relationship between *vgll3*L* and catch date, fitted with a GAM, whereas the grey area around the line illustrates the uncertainty of the fitted relationship. The data points represent the *vgll3* genotype of individual samples. The points are jittered on the y-axis to aid figure interpretation. TK refers to Tornio-Kalix.


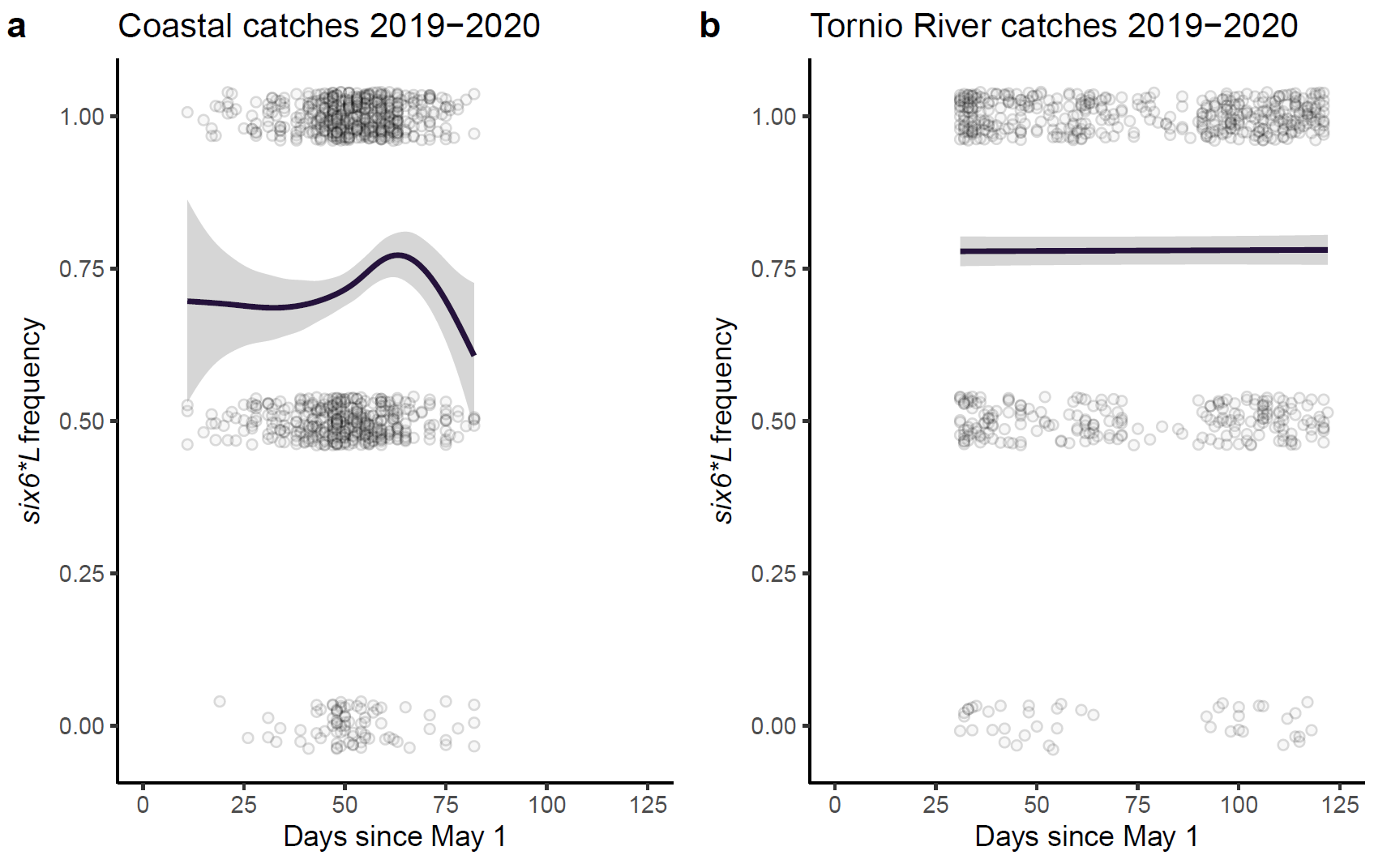


**Figure S5.** Frequency of *six6*L* in wild salmon from **a** coastal catches, and in **b** Tornio River catches during 2019-2020, summarised across all areas. The line depicts a relationship between *six6*L* and catch date, fitted with a GAM, whereas the grey area around the line illustrates the uncertainty of the fitted relationship. The data points represent the *six6* genotype of individual samples. The points are jittered on the y-axis to aid figure interpretation. TK refers to Tornio-Kalix.

**
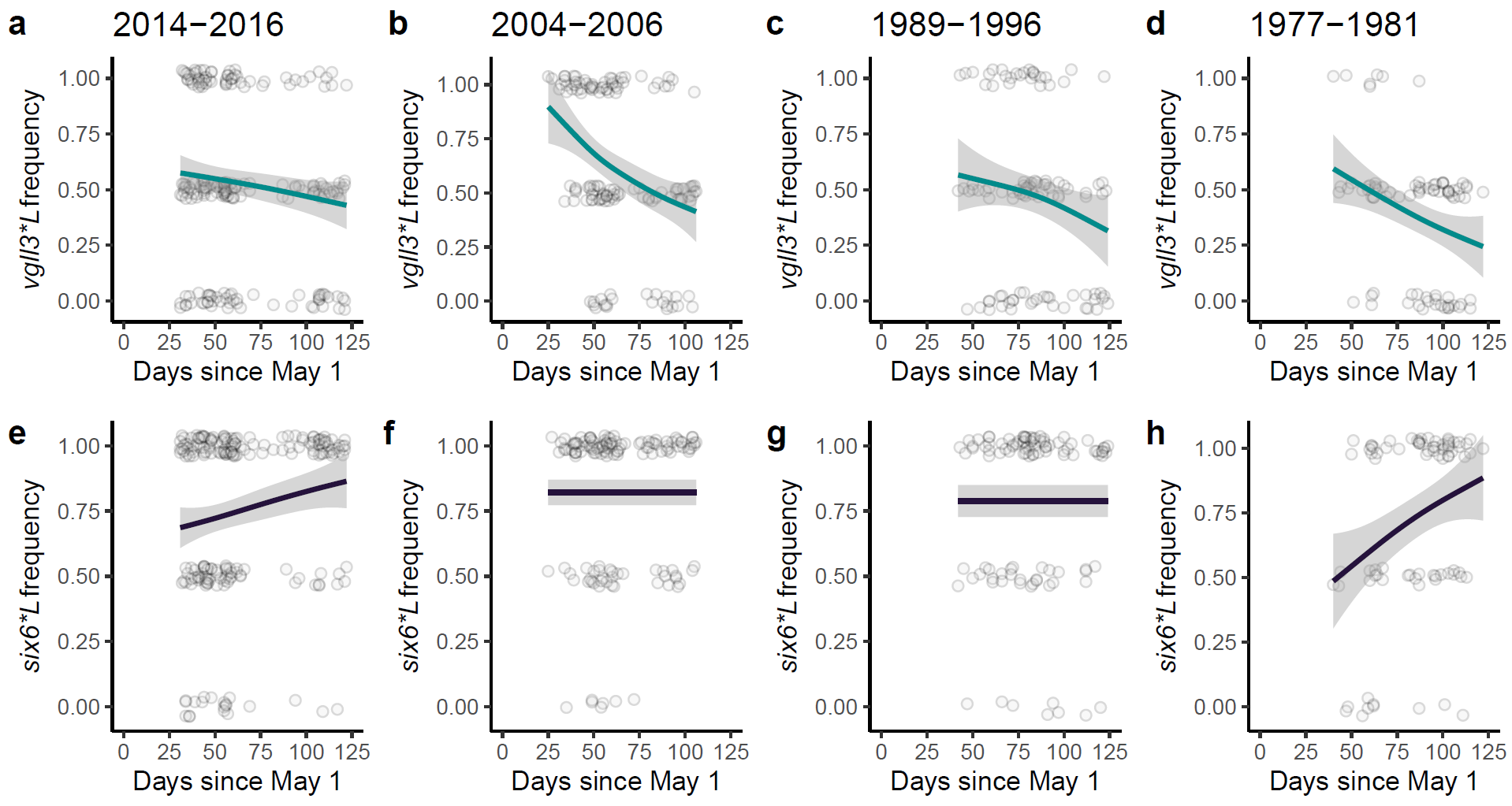
**

**Figure S6.** Allele frequencies of *vgll3*L* and *six6*L* in Tornio River salmon catches during **a/e** 2014-2016, **b/f** 2004-2006, **c/g** 1989-1996, and **d/h** 1977-1981. The lines depict a relationship between *vgll3*L* or *six6*L* and catch date, fitted with a GAM, whereas the grey area around the lines illustrates the uncertainty of the fitted relationship. The data points represent the *vgll3* or *six6* genotype of individual samples. The points are jittered on the y-axis to aid figure interpretation.


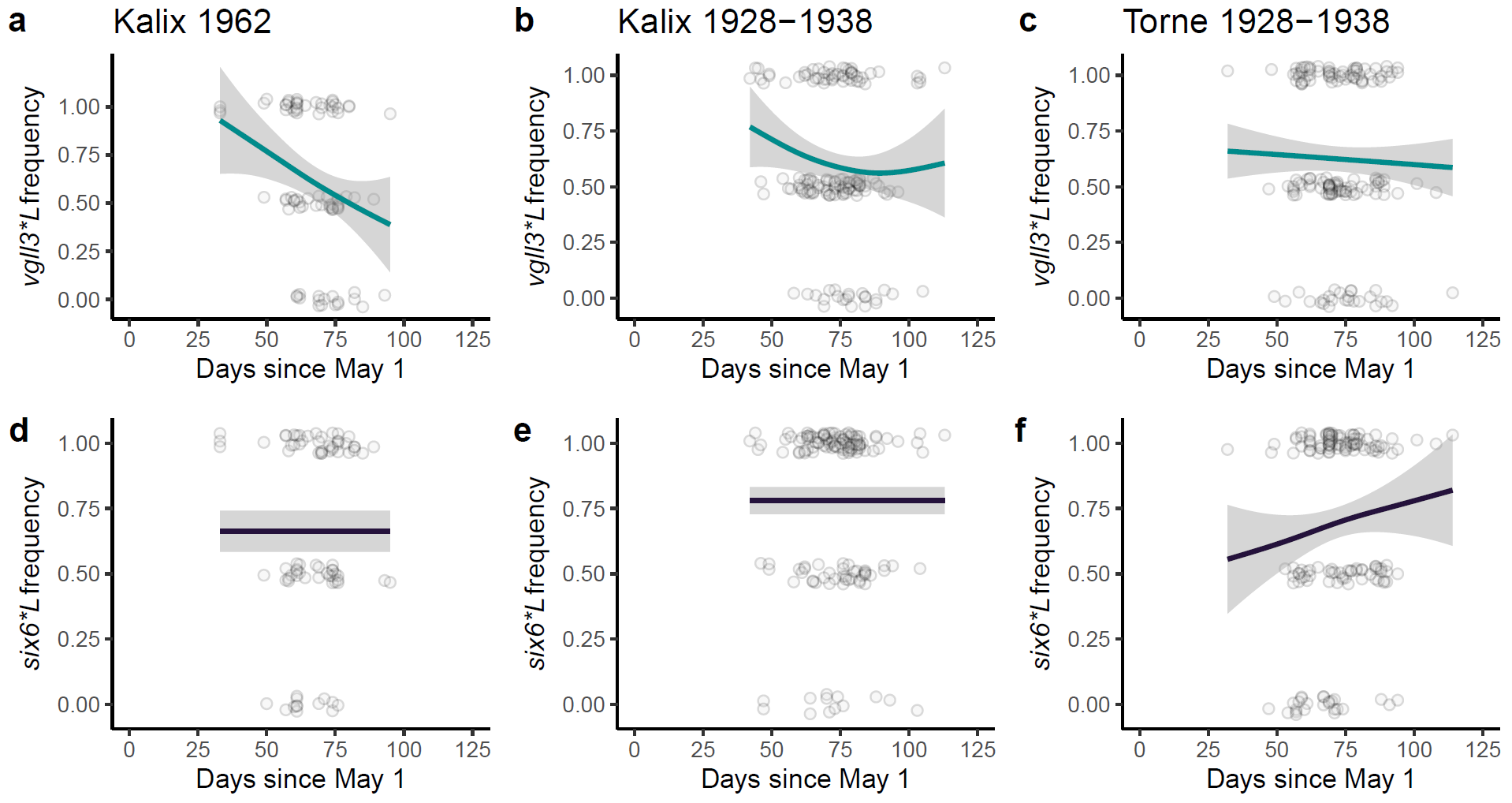


**Figure S7.** Allele frequencies of *vgll3*L* and *six6*L* in river salmon catches during **a/d** 1962 (Kalix), **b/e** 1928-1938 (Kalix), and **c/f** 1928-1938 (Torne). The lines depict a relationship between *vgll3*L* or *six6*L* and catch date, fitted with a GAM, whereas the grey area around the lines illustrates the uncertainty of the fitted relationship. The data points represent the *vgll3* or *six6* genotype of individual samples. The points are jittered on the y-axis to aid figure interpretation.


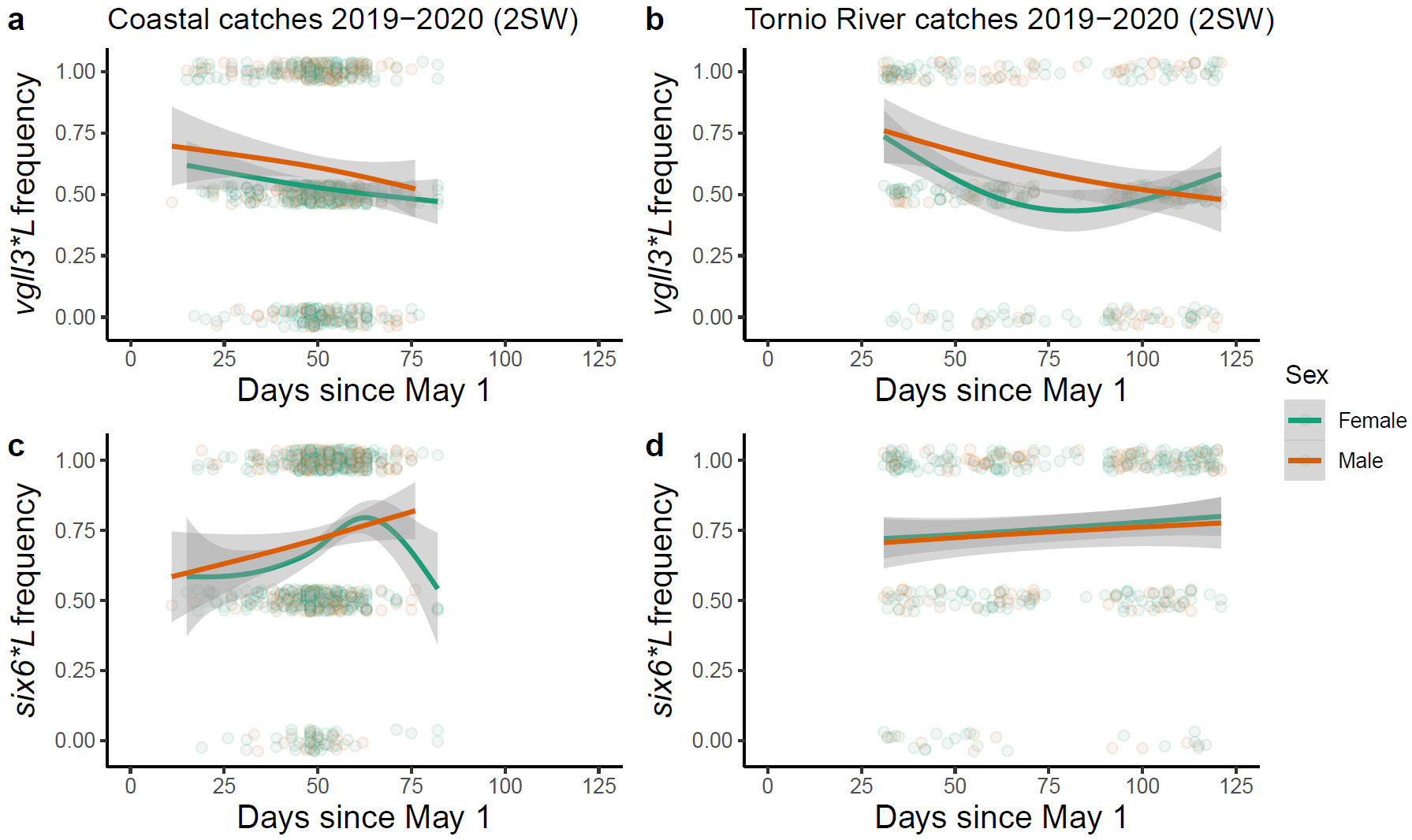


**Figure S8.** Allele frequencies of **a, b** *vgll3*L* in coastal and Tornio River wild salmon catches, respectively, during 2019-2020, and **c, d** *six6*L* in coastal and Tornio River wild salmon catches, respectively, during 2019-2020, in two-sea-winter (2SW) salmon only. The lines depict a relationship between *vgll3*L* or *six6*L* and catch date, fitted with a GAM, whereas the grey area around the lines illustrates the uncertainty of the fitted relationship. The data points represent the *vgll3* or *six6* genotype and sex of individual samples. The points are jittered on the y-axis to aid figure interpretation.


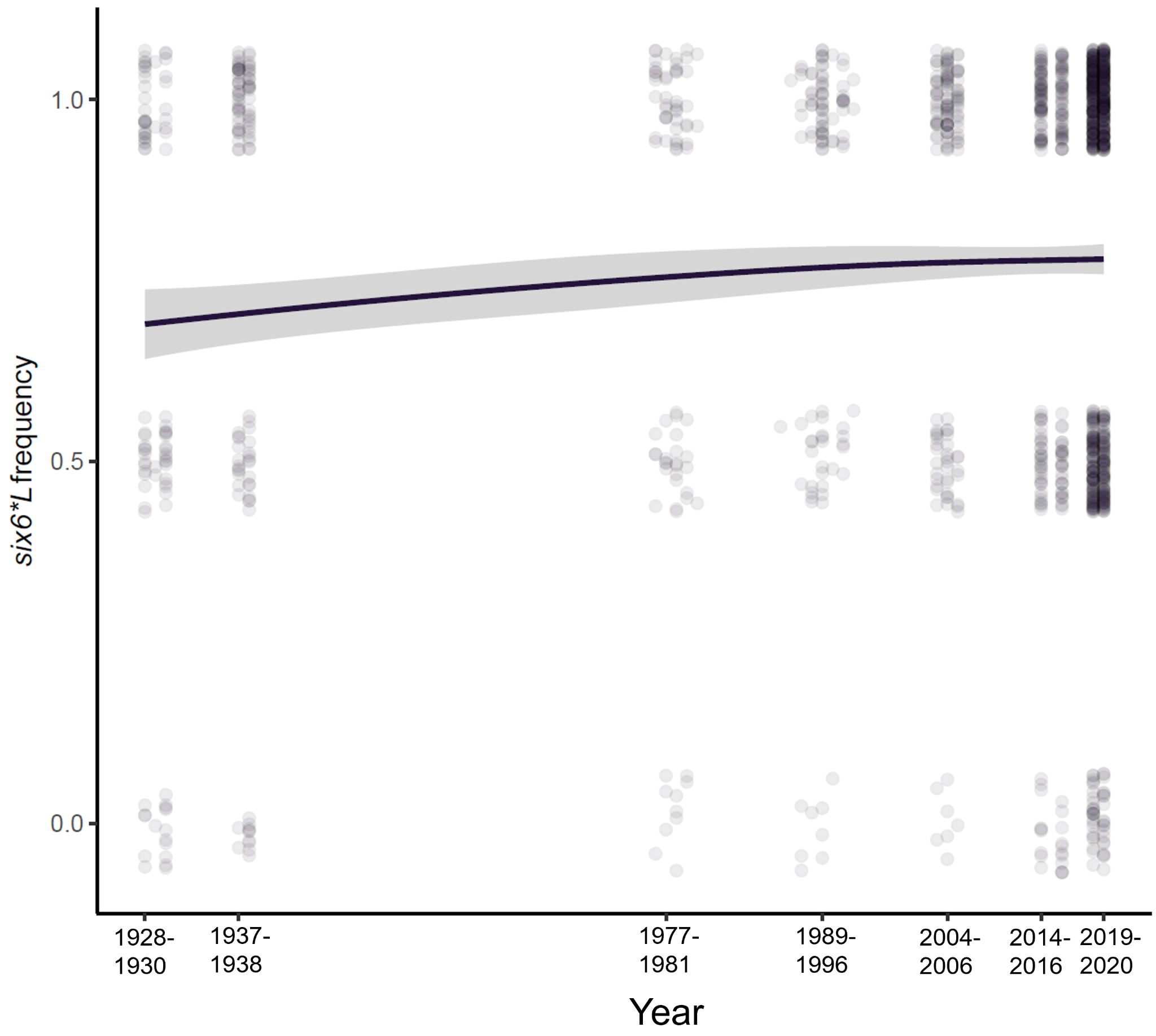


**Figure S9.** Long-term changes in *six6*L* allele frequency in the Tornio/Torne River salmon catches from 1928 to 2020. The line depicts a relationship between *six6*L* and sampling year, fitted with a GAM, whereas the grey area around the lines illustrates the uncertainty of the fitted relationship. The data points represent the *six6* genotype of individual samples. The points are jittered on the y-axis to aid figure interpretation.


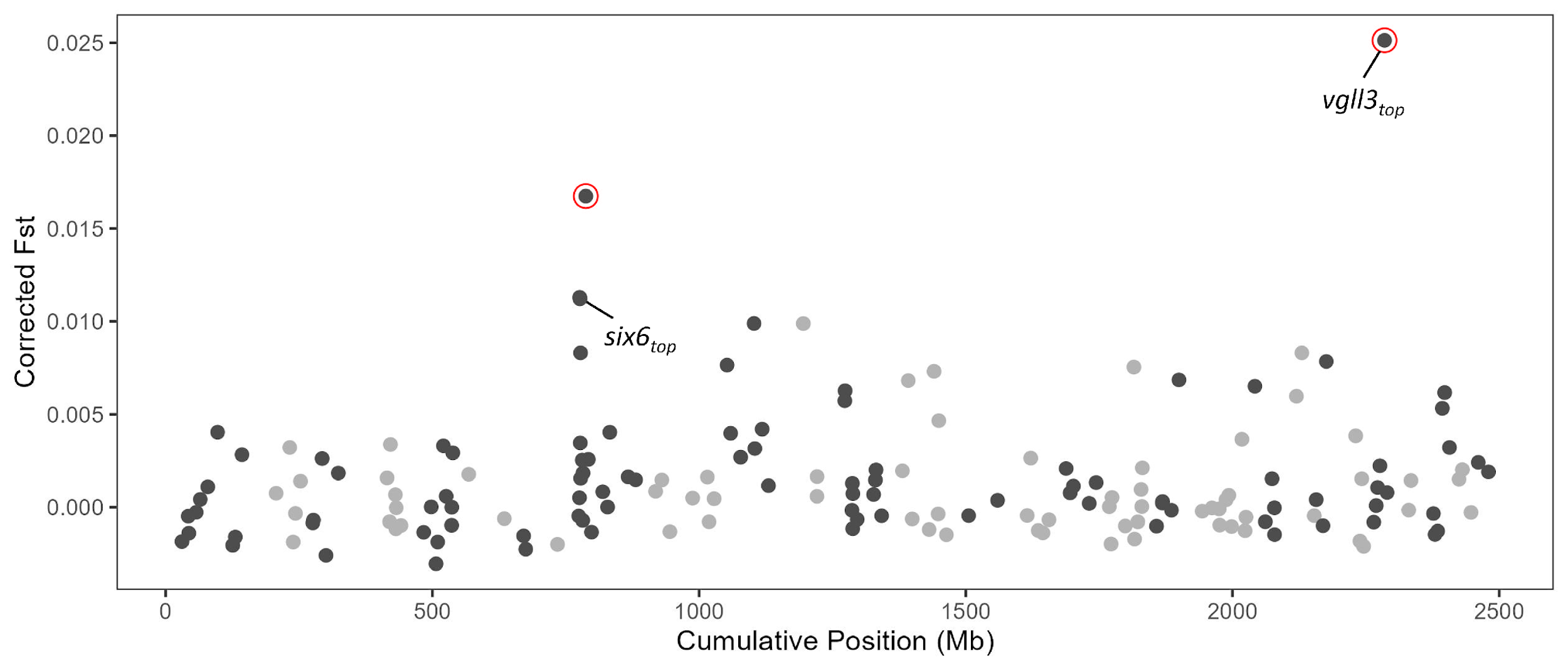


**Figure S10.** Results of the OutFLANK analysis across Tornio River fishery samples collected at 7 time points between 1928 and 2020. SNPs are ordered along the x-axis by their cumulative position on the *S. salar* genome (Ssal_v3.1), with different grey shades indicating different chromosomes. F_ST_ corrected for sample size is shown on the y-axis. Significant outliers (q < 0.05) are circled in red.

**Supplementary Methods and Results**

**Development of a SNP marker panel for genetic monitoring and stock identification of northern Baltic Atlantic salmon**

1. **Methods**

**1.1 Genetic baseline dataset**

We used information from earlier studies (e.g. Whitlock *et al.*, 2018) to determine which Baltic salmon stocks were expected to contribute fish to our focal salmon harvests from the northern Baltic Sea region (fishing areas shown in Figure 2 in main text). On the basis of this information, we gathered 688 baseline samples from four wild river stocks (Tornio, Kalix, Simo, and Råne Rivers) and five stocks of reared origin (Tornio, Simo, Ii, Oulu and Lule stocks) (Figure SX1, Table SX1).

The wild baseline samples were taken from electrofished juveniles (parr; n = 499), or trapped smolts (n = 34), while the broodstock samples were from adults (n = 155). The tissue material collected was either fin clips preserved in 95% ethanol or scale samples stored dry in paper envelopes. We extracted DNA from the baseline samples using the QIAGEN DNAamp Mini Kit method, the QuickExtract™ DNA Extraction Solution (Lucigen), or a salt extraction method (Aljanabi & Martinez, 1997).


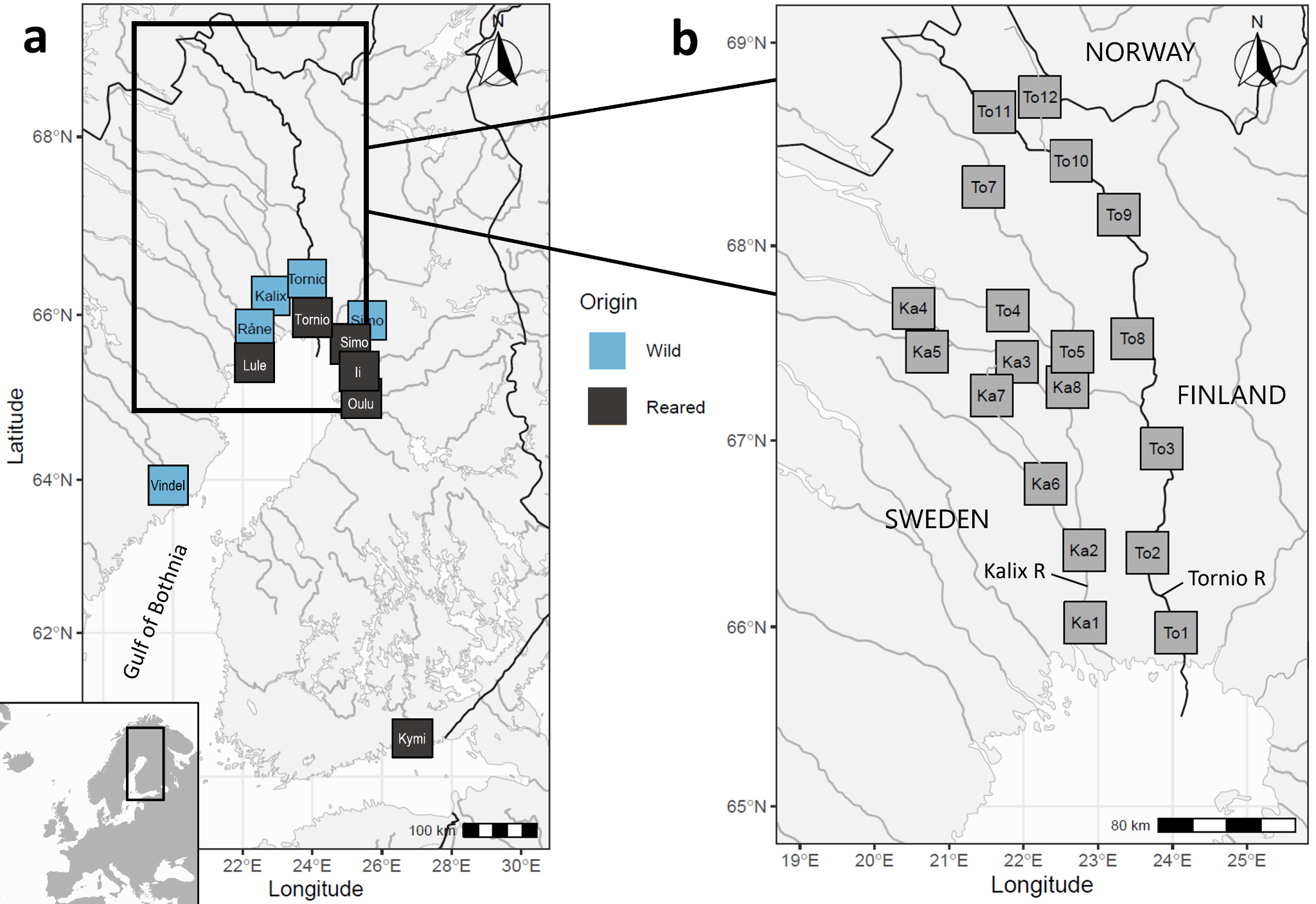


**Figure SX1.** The geographic origin of **a** all salmon stocks in the genetic baseline used in developing and/or using the SNP panel, and **b** sampling sites of wild baseline samples in the Tornio-Kalix River system. The darker lines depict national borders, including the Tornio/Torne River that flows on the border of Finland and Sweden.

**Table SX1.** Samples used in the development and use of a SNP marker panel for genetic monitoring and stock identification of northern Baltic Atlantic salmon. Cohort refers to the year of fertilisation of the hatchery broodstocks. GSI = Genetic stock identification.

| **Origin** | **Year** | **Life stage** | **n^a^** | **n^b^** | **Sites electrofished (n)** | Origin | **Used for** |
| --- | --- | --- | --- | --- | --- | --- | --- |
| Ii River hatchery^c^ | 2009 (cohort) | Adult | 18 | 11 |  | Reared | SNP panel development only |
|  | 2013 (cohort) | Adult | 32 | 21 |  |  |  |
| Oulu River hatchery^c^ | 2007 (cohort) | Adult | 12 | 10 |  | Reared | SNP panel development only |
|  | 2010 (cohort) | Adult | 12 | 11 |  |  |  |
| Simo River hatchery^c^ | 2011 (cohort) | Adult | 18 | 18 |  | Reared | SNP panel development only |
| Tornio River hatchery^c^ | 2004 (cohort) | Adult | 13 | 12 |  | Reared | SNP panel development only |
|  | 2007 (cohort) | Adult | 13 | 12 |  |  |  |
|  | 2012 (cohort) | Adult | 13 | 12 |  |  |  |
| Kymi River^d^ | 2017 | Adult | 20 | 19 |  | Reared | GSI only |
| Lule River^c^ | 2018-2019 | Adult | 24 | 23 |  | Reared | SNP panel development only |
| Råne River^c^ | 2012, 2019 | Parr (2012), smolts (2019) | 34 | 31 |  | Wild | GSI, SNP panel development |
| Simo River^c^ | 2019 | Parr | 122 | 111 | 32 | Wild | GSI, SNP panel development |
| Tornio River^c^ | 2012-2014 | Parr | 222 | 208 | 34 | Wild | GSI, SNP panel development |
| Kalix River^c^ | 2012-2015 | Parr | 155 | 153 | 18 | Wild | GSI, SNP panel development |
| Vindel River^d^ | 2015 | Smolt | 22 | 22 |  | Wild | GSI only |

^a^ Total number of genotyped samples, ^b^ Samples in the filtered dataset, ^c^ Genotyped with 60,252 SNP markers, ^d^ Genotyped with 229 SNP markers.

**1.1.1 Genotyping and quality control**

We genotyped the baseline samples with a custom single nucleotide polymorphism (SNP) microarray (Affymetrix Axiom) containing 60,252 markers with known positions on the Atlantic salmon genome (CIGENE; Norwegian University of Life Sciences, Norway). We included a set of 13 replicate individuals per genotyping run to control for batch effects in genotype calling among runs.

Using PLINK 1.90 (Chang *et al.*, 2015), we subsequently applied the following quality control steps: we removed SNPs that i) did not map to an assembled Atlantic salmon chromosome, ii) showed any mismatches within replicate individuals across genotyping runs, iii) had > 10% missing genotypes, or iv) a minor allele frequency (MAF) < 0.05. We also filtered out a set of SNPs that were identified as having possible technical genotyping problems on the basis of strong deviations from Hardy-Weinberg equilibrium (p < 0.00001) within samples from the lower parts of the Tornio-Kalix system (n = 174) which is known *a priori* not to contain strong substructure (Miettinen *et al.*, 2021). Finally, we removed individuals with > 10% missing genotypes. After the quality control filtering, 37,255 SNPs remained in the dataset.

Family sampling in the baseline can bias population structure analysis and genetic stock identification (GSI) (e.g. Östergren, Palm, Gilbey, & Dannewitz, 2020). We examined our baseline for putative full siblings by splitting the dataset by sampling site (for the Tornio-Kalix samples) or river/broodstock (for all other samples), reapplying the MAF < 0.05 filter, using PLINK 1.90’s *--genome* function to estimate identity-by-state (IBS) between each pair of individuals, and then used a PI_HAT (pairwise identity-by-descent value) threshold of 0.35 to identify putative full siblings, and retained only one individual from any full-sib group in the baseline. After the quality control filtering and full-sib removal, 633 individuals were retained in the genetic baseline.

**1.1.2 Genetic population structure**

We used a linkage disequilibrium (LD)-pruned version of our filtered SNP array dataset (21,547 SNPs; PLINK 1.90 command *--indep-pairwise 50 5 0.5*) to explore population structure of the baseline. We initially explored genetic structure by performing a multidimensional scaling analysis on the genome-wide identity-by-state pairwise distances (PLINK 1.90), and visualising this graphically with *ggplot2* (Wickham 2016) (Figure SX2). We also used ADMIXTURE version 1.3.0 (Alexander, Novembre, & Lange, 2009) and inferred the most suitable number of genetic clusters from five-fold cross-validation error rates and visual inspection of the results (Figure SX3).

**1.2 Development of GT-seq SNP marker panel**

**1.2.1 Candidate SNP selection**

We used a preliminary version of the microarray baseline (not including samples from Tornio-Kalix sites To2, To9, To10 and To11) to identify a panel of candidate SNPs to be used in GSI. This dataset (“Baltic baseline”) included 418 individuals. Candidate SNPs were identified as follows:

i) We based our GSI panel on an existing, validated genotyping-by-sequencing panel developed for GSI in the Teno/Tana River of Finland and Norway (Johansson *et al.,* in prep). We filtered this “Teno” panel to exclude SNPs with a MAF < 0.05 and a global FST < 0.01 across the Baltic baseline populations. This panel included the SNPs *vgll3_top_*, *six6_top_* and a SNP on the male-specific *sdY* gene, as well as SNPs linked to six other genes of possible adaptive significance in Teno salmon, and we retained all of these for the Baltic SNP panel developed here. Ninety-five Teno panel SNPs were taken forward into the candidate Baltic SNP panel.

ii) For identification and testing of additional candidate SNPs from the microarray, we divided the baseline into two, partly overlapping, datasets. The “choosing” dataset was used to identify candidate SNPs. The “testing” dataset was used to test the assignment efficacy of the SNPs while partly avoiding ascertainment bias that would arise by selecting SNPs and testing their assignment using the same set of individuals.

The “choosing” dataset included two-thirds of the individuals from the larger baseline samples (lower Tornio-Kalix, upper Tornio-Kalix, wild Simo River) and all individuals from the rest of the baseline samples (not split due to smaller sample sizes) except Råne. Råne was removed from the “choosing” dataset because it is a likely bottlenecked population strongly distinct from the rest of the baseline and would therefore bias F_ST_ estimation during the SNP selection process, while being easily identified with any set of SNPs. The “testing” dataset contained the remaining third of the larger baseline samples, plus all other baseline samples including Råne.

iii) We filtered the “choosing dataset” baseline to retain only highly variable SNPs (MAF > 0.4).

iv) We ranked the remaining SNPs by global F_ST_ across the samples in the “choosing dataset” and added the top 90 of these to the SNPs retained from the Teno panel.

v) We tested the efficacy of this new SNP set (n = 185) for GSI in the “testing dataset”, using a “leave-one-out” population re-assignment approach implemented in the R package *rubias* (R Core Team 2021; Moran & Anderson, 2019). We used the proportion of baseline individuals re-assigned to their correct population as a measure of SNP panel performance.

vi) We applied an iterative approach of a) examining the *rubias* results to identify particular sets of populations with high cross-assignment of individuals between them, b) subsetting these populations from the “choosing dataset” and ranking SNPs by their global F_ST_ across these populations, c) adding various numbers of top-ranked SNPs to the existing SNP set, and d) re-testing the assignment power of the new SNP panels within the “testing dataset”. We added SNPs to improve assignment to the hatchery stocks only if they did not compromise the power of the SNP panel to discriminate wild populations.

vii) Finally, we added the following SNPs to the candidate SNP panel for purposes other than fisheries GSI: a) 55 SNPs that were identified via a preliminary outlier analysis of the genome-wide dataset as potentially being under local selection within the Tornio-Kalix river system, for possible monitoring of adaptive genetic change, and b) eight SNPs in our baseline that were used in a recent study of Baltic salmon populations from Swedish rivers (Östergren *et al.*, 2021), for possible comparison across studies.

viii) We identified SNPs that were in strong linkage disequilibrium with one another in our candidate dataset by using PLINK 1.90 (command *--r2 inter-chr*). We assessed this separately for each of the two largest population groups in our dataset (i.e. the lower and upper Tornio-Kalix), and retained only one SNP from a pair that had a pairwise R^2^ value > 0.4 in both groups. Additionally, we retained only one SNP from a pair that were physically < 100 kb apart. During these filtering steps, we preferentially retained SNPs in the established Teno/Tana GSI panel.

**1.2.2 GT-seq primer design**

The final set of candidate SNPs was taken forward to genotyping-by-sequencing assay development (GT-seq, Campbell, Harmon, & Narum, 2015). We designed primers to amplify a 70-110bp DNA sequence surrounding each target SNP using *BatchPrimer3* (You *et al.*, 2008), supplying a narrow range of possible reaction parameters (default parameters except for product size: min 70bp, opt 80bp, max 110bp; primer size: 18-35bp; primer tm: 60.5-62.5°C; max tm difference: 2°C, primer GC: 40-60%) to ensure successful multiplexing.

**1.2.3 GT-seq genotyping and genetic sexing**

For both initial testing and subsequent genotyping rounds, the primer pairs for all the SNPs were combined into one or two multiplex mixes, which were used to amplify the target sequences in PCR reactions using a Qiagen Multiplex PCR kit. Each 9 µl PCR reaction contained 3 µl primers (each at 0.015-0.06 µM), 4.5 µl Multiplex Mix, 1.5 µl DNA with the following thermocycling conditions: 15 min at 95°C followed by 13 cycles of 30 s at 95°C, 1 min at 58°C and 45 s at 72°C, followed by 15 cycles of 30 s at 95°C, 1 min at 62°C and 45 s at 72°C, and a final step of 3 min at 72°C. PCR products were tagged with individual-specific combinatorial barcodes, pooled, and single-end sequenced at the DNA Sequencing and Genomics Laboratory, Institute of Biotechnology, University of Helsinki, using Illumina iSeq, MiSeq or NextSeq platforms with Illumina chemistry. The obtained sequences were split by barcode, the reverse adaptor sequence and any downstream subsequent sequence read was trimmed using *cutadapt* 3.5 (Martin, 2011), and the trimmed reads were aligned to the Atlantic salmon reference genome using *bwa mem* with default parameters (bwa 0.7.17, Li, 2013). The genotypes at the target SNP sites were called from the sorted bam files using *bcftools mpileup* followed by *call* (Danecek *et al.*, 2021), applying the consensus caller and outputting genotype quality flags with the genotypes. Reads from repeat sequencing of the same individuals were combined into single files for SNP calling.

We genetically sexed all individuals by comparing the sequencing coverage across the male-specific *sdY* to the mean sequencing coverage across the autosomal SNPs. To avoid false identifications of females as males, we classified individuals as females if the ratio of their *sdY* coverage compared to autosomal SNPs was < 0.2. Individuals with a ratio between 0.2 and 0.8 were considered as unassigned, and above 0.8 as male.

**1.2.4 GT-seq panel optimization**

We carried out four rounds of multiplex PCR optimisation to minimise primer dimers, improve sequencing coverage, and remove poorly performing SNPs. The first three rounds used the same 46 test samples, 24 of which had previously been genotyped with the SNP microarray. We performed additional multiplex optimisation after the first full genotyping run, which included another 91 samples that had been genotyped with the microarray.

For the first round, all primer pairs were combined into a single multiplex pool and an in-house Linux script was applied to the trimmed sequence output to identify and count primer-dimer reads. Primer pairs were then separated into two multiplex pools with the aim of minimising dimer formation within each pool. The subsequent optimization rounds were used to re-adjust primer pool composition and the relative concentrations of individual primer pairs, and to remove SNPs with >10% mismatches between the GT-seq genotypes and microarray genotypes or continued poor amplification success.

1. **Results**

**2.1 Genotyping success of SNP array**

Twenty-two of the samples failed at the SNP microarray genotyping stage (dish QC score < 0.95 and a call rate < 0.97), resulting in 666 samples in the raw microarray dataset. After quality control filtering, full-sib removal and LD pruning 21,390 SNPs and 633 individuals were used for the population structure analysis.

**2.2 Genetic population structure**

According to cross-validation (CV) error from ADMIXTURE, the optimal number of ancestral genetic clusters (*K)* in the dataset was 6. However, *K* = 9 also had high likelihood and was most in line with geography, as it largely separated the following locations and stocks: 1) lower Tornio-Kalix wild and Tornio hatchery, 2) upper Tornio-Kalix wild, 3) upper Lainio wild, 4) Ängesån wild, 5) Simo wild and Simo hatchery, 6) Råne wild, 7) Ii hatchery, 8) Oulu hatchery, and 9) Lule sea-ranched stock (Figures SX2-SX3). We used these nine clusters as the genetic reporting units for subsequent SNP panel design.

**
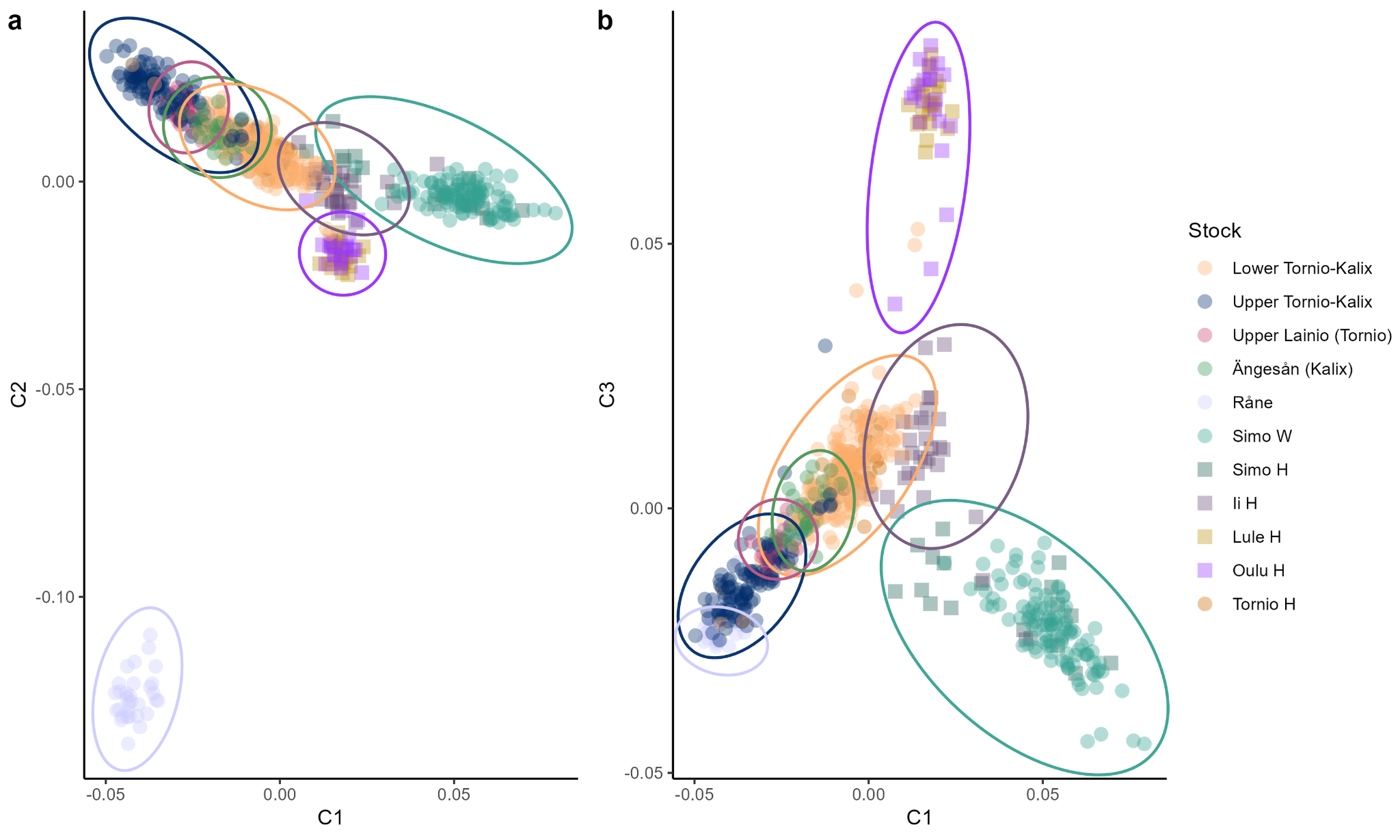
**

**Figure SX2.** Multidimensional scaling (MDS) plots visualising the genetic population structure of the genetic baseline of Baltic salmon populations in this study (n = 633), using 21,547 SNPs. Each point represents an individual sample, and their distribution on the first two MDS axes depicts their genetic distance from each other. The squares describe hatchery individuals, and the circles wild individuals. **a** MDS axes C1 and C2, and **b** MDS axes C1 and C3. The ellipses describe the clusters identified by ADMIXTURE (note that Oulu H and Lule H are grouped together here).


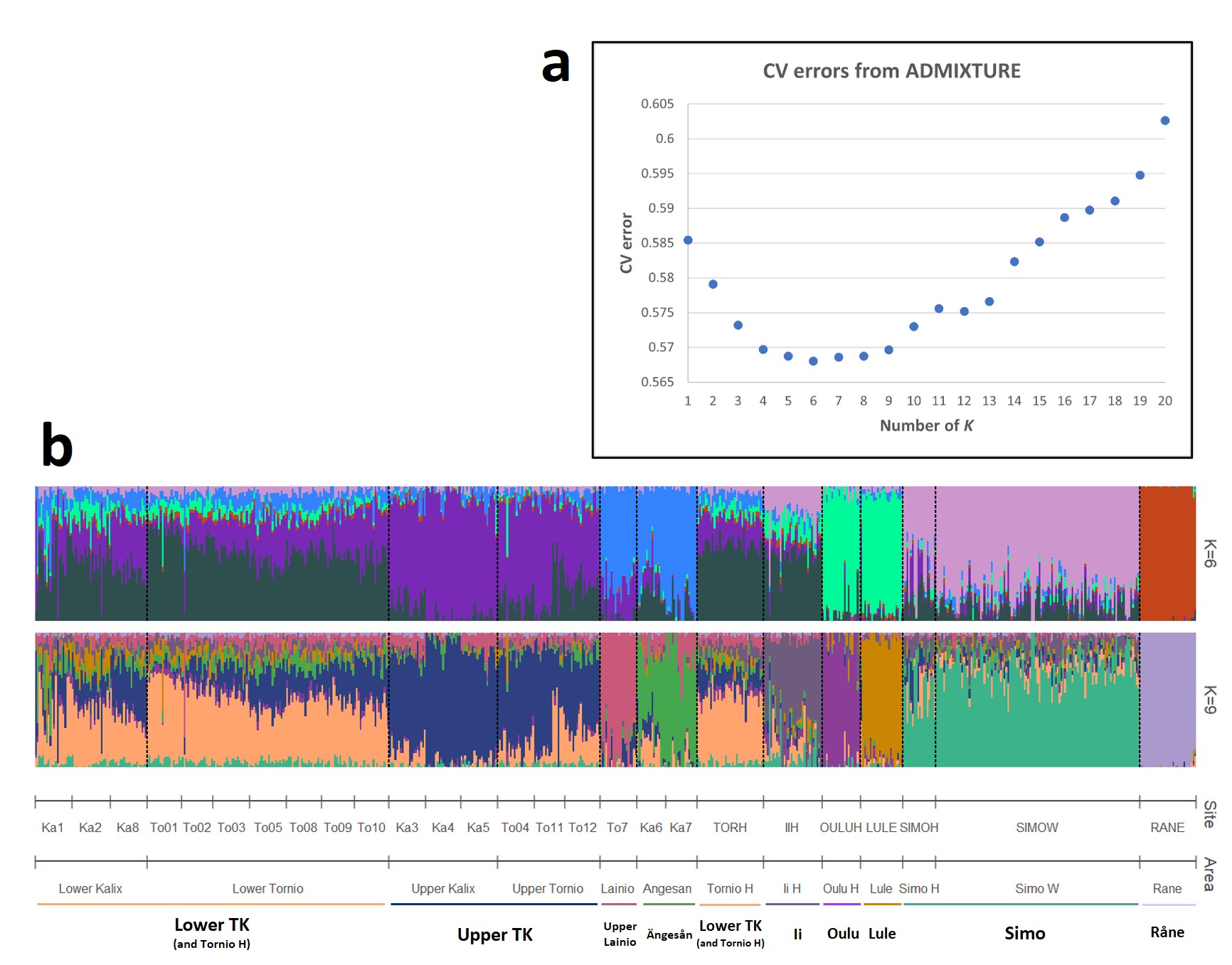


**Figure SX3.** **a** Cross-validation (CV) error estimates from ADMIXTURE, to determine the optimal number of genetic clusters in the genetic baseline dataset of northern Baltic salmon stocks (n = 633 samples, n = 21,549 SNPs). **b** ADMIXTURE plot showing the optimal *K* (number of genetic clusters) according to minimal cross-validation (CV) error (*K* = 6), and the biologically most meaningful number of clusters (*K* = 9; outlined on the bottom row) in the genetic baseline dataset of northern Baltic salmon stocks (n = 633 samples, n = 21,547 SNPs). Each colour represents a different cluster.

**2.3 GT-seq SNP panel development**

The final candidate SNP set comprised 309 SNPs. Fifty-one of these failed the primer design process, resulting in 258 SNPs that were taken forward to testing. During the optimisation rounds, an additional 29 SNPs were removed due to inescapable primer dimer formation, mismatches between genotypes from the GT-seq and the microarray genotyping, or failure of amplification in the optimisation runs. This left a final panel of 229 SNPs (including *vgll3_top_*, *six6_top_* and *sdY)* that were used in all GT-seq genotyping runs (Table S5).

**References**

Alexander, D. H., Novembre, J., & Lange, K. (2009). Fast model-based estimation of ancestry in unrelated individuals. Genome Research, 19(9), 1655–1664. https://doi.org/10.1101/gr.094052.109

Aljanabi, S. M., & Martinez, I. (1997). Universal and rapid salt-extraction of high quality genomic DNA for PCR-based techniques. Nucleic Acids Research, 25(22), 4692–4693.

Campbell, N. R., Harmon, S. A., & Narum, S. R. (2015). Genotyping-in-Thousands by sequencing (GT-seq): A cost effective SNP genotyping method based on custom amplicon sequencing. Molecular Ecology Resources, 15(4), 855–867. https://doi.org/10.1111/1755-0998.12357

Chang, C. C., Chow, C. C., Tellier, L. C. A. M., Vattikuti, S., Purcell, S. M., & Lee, J. J. (2015). Second-generation PLINK: Rising to the challenge of larger and richer datasets. GigaScience, 4(1), 1–16. https://doi.org/10.1186/s13742-015-0047-8

Danecek, P., Bonfield, J. K., Liddle, J., Marshall, J., Ohan, V., Pollard, M. O., … Davies, R. M. (2021). Twelve years of SAMtools and BCFtools. GigaScience, 10(2), 1–4. https://doi.org/10.1093/gigascience/giab008

Li, H. (2013). Aligning sequence reads, clone sequences and assembly contigs with BWA-MEM, 00(00), 1–3. Retrieved from http://arxiv.org/abs/1303.3997

Martin, M. (2011). Cutadapt removes adapter sequences from high-throughput sequencing reads. EMBnet.Journal 17.1, 10–12.

Miettinen, A., Palm, S., Dannewitz, J., Lind, E., Primmer, C. R., Romakkaniemi, A., … Pritchard, V. L. (2021). A large wild salmon stock shows genetic and life history differentiation within, but not between, rivers. Conservation Genetics, 22(1), 35–51. https://doi.org/10.1007/s10592-020-01317-y

Moran, B. M., & Anderson, E. C. (2019). Bayesian inference from the conditional genetic stock identification model. Canadian Journal of Fisheries and Aquatic Sciences, 76(4), 551–560. https://doi.org/10.1139/cjfas-2018-0016

Östergren, J., Palm, S., Gilbey, J., & Dannewitz, J. (2020). Close relatives in population samples: Evaluation of the consequences for genetic stock identification. Molecular Ecology Resources, 20(2), 498–510. https://doi.org/10.1111/1755-0998.13131

Östergren, J., Palm, S., Gilbey, J., Spong, G., Dannewitz, J., Königsson, H., … Vasemägi, A. (2021). A century of genetic homogenization in Baltic salmon – evidence from archival DNA. Proceedings of the Royal Society B: Biological Sciences 288:20203147. https://doi.org/10.1098/rspb.2020.3147

R Core Team (2021). R: A language and environment for statistical computing. R Foundation for Statistical Computing, Vienna, Austria. <https://www.R-project.org/>.

Wickham H (2016). ggplot2: Elegant graphics for data analysis. Springer-Verlag.

You, F. M., Huo, N., Gu, Y. Q., Luo, M. C., Ma, Y., Hane, D., … Anderson, O. D. (2008). BatchPrimer3: A high throughput web application for PCR and sequencing primer design. BMC Bioinformatics, 9, 1–13. https://doi.org/10.1186/1471-2105-9-253
